# SHIPS: Spectral Hierarchical Clustering for the Inference of Population Structure in Genetic Studies

**DOI:** 10.1371/journal.pone.0045685

**Published:** 2012-10-12

**Authors:** Matthieu Bouaziz, Caroline Paccard, Mickael Guedj, Christophe Ambroise

**Affiliations:** 1 Department of Biostatistics, Pharnext, Paris, France; 2 Statistics and Genome Laboratory, University of Evry Val d'Essonne, UMR CNRS 8071 - USC INRA, Evry, France; Aarhus University, Denmark

## Abstract

Inferring the structure of populations has many applications for genetic research. In addition to providing information for evolutionary studies, it can be used to account for the bias induced by population stratification in association studies. To this end, many algorithms have been proposed to cluster individuals into genetically homogeneous sub-populations. The parametric algorithms, such as Structure, are very popular but their underlying complexity and their high computational cost led to the development of faster parametric alternatives such as Admixture. Alternatives to these methods are the non-parametric approaches. Among this category, AWclust has proven efficient but fails to properly identify population structure for complex datasets. We present in this article a new clustering algorithm called Spectral Hierarchical clustering for the Inference of Population Structure (SHIPS), based on a divisive hierarchical clustering strategy, allowing a progressive investigation of population structure. This method takes genetic data as input to cluster individuals into homogeneous sub-populations and with the use of the gap statistic estimates the optimal number of such sub-populations. SHIPS was applied to a set of simulated discrete and admixed datasets and to real SNP datasets, that are data from the HapMap and Pan-Asian SNP consortium. The programs Structure, Admixture, AWclust and PCAclust were also investigated in a comparison study. SHIPS and the parametric approach Structure were the most accurate when applied to simulated datasets both in terms of individual assignments and estimation of the correct number of clusters. The analysis of the results on the real datasets highlighted that the clusterings of SHIPS were the more consistent with the population labels or those produced by the Admixture program. The performances of SHIPS when applied to SNP data, along with its relatively low computational cost and its ease of use make this method a promising solution to infer fine-scale genetic patterns.

## Introduction

Population structure relates the genetic heterogeneity that exists between individuals of a population. This heterogeneity is a natural phenomenon resulting from biological and evolutionary processes such as for instance natural selection, genetic drift, populations migrations or mating processes [1]. These phenomena lead in time to sub-populations genetically differing with regard to the frequency of certain alleles. For the same reasons, disease prevalences or allele penetrances may vary between such groups. These systematic differences between sub-populations can be more or less important. The most identifiable are found between ethnic and/or geographically distant groups.

Identifying the underlying structure of populations is often of use for genetic research. It allows the study of evolutionary relationships between populations as well as learning about their demographic histories [2]–[6].

Such analyses are also of a great interest for population-based genetic studies such as Genome-Wide Association Studies (GWASs). Notwithstanding the widespread usage of GWASs, their findings have been criticized partly because they are vulnerable to population stratification. This corresponds to the bias induced in situations where the studied populations are genetically heterogeneous and the sampling of cases and controls is imbalanced between the various ancestries. Population stratification is known to lead to finding spurious associations or to missing genuine ones [7]–[11]. Inferring the structure of the populations can therefore be helpful to identify whether there is indeed a structure or to define homogeneous clusters of individuals that can later be used to correct the association test and account for stratification.

Two major strategies have been developed to infer the structure of the populations that are parametric model-based clustering and non-parametric clustering. Model-based clustering approaches make numerous assumptions on the genetic data and use statistical inference methods to assign individuals to sub-populations. Many of these parametric approaches exist such as for instance Structure [5], Admixture [12], [13], BAPS [14] or FRAPPE [15]. These parametric methods are more commonly used to infer population structure. It has however been pointed out that they have some drawbacks such as the complexity of the underlying statistical models and of the assumptions that have to been made on the data. Also, the program Structure is known to have a very high computational cost. Non-parametric approaches have the advantage over parametric ones of making fewer assumptions on the data. For example most of these methods do not assume the Hardy-Weinberg equilibrium between genetic markers. In addition, such approaches involve few parameters to be estimated [16]. The main non-parametric methods are Awclust [17] using a distance-based hierarchical clustering or ipPCA [1] using iterative principal component analysis (PCA). It is also possible to apply clustering algorithms, such as a Gaussian mixture model-based clustering, to the principal components resulting from a PCA applied to genetic data [6]. We refer to this particular method as PCAclust in the following.

We propose in this paper a novel non-parametric distance-based clustering approach based on a divisive hierarchical clustering method. Our method is based on the idea that it might not be possible to uncover all of the structure in the data when applying a clustering algorithm just once. Fine population structures may not be detected as the corresponding sub-populations are hidden within the major sub-populations detected by the first run of the algorithm.

We therefore implemented a robust statistical framework to iteratively apply a clustering algorithm to the data and so analyze in depth the genetic patterns of the studied populations. This corresponds to a divisive hierarchical clustering strategy. Based on a pairwise distance matrix, the algorithm progressively divides the original population in two sub-populations by the use of a spectral clustering algorithm. The process is then iterated in each of the two sub-populations and so on. This leads to the construction of a binary tree, where each node represents a group of individuals. To determine the final clusters, a tree pruning procedure and an estimation of the optimal number of clusters are applied. In such an approach, both the final clustering of the individuals and the number of clusters are estimated by the method. We call our method ‘Spectral Hierarchical clustering for the Inference of Population Structure’ (SHIPS).

We present in this article the SHIPS algorithm along with several applications to SNP datasets. We consider five scenarios of simulated population structures. The software Genome [18] was used to simulate these data of increasing complexity. We also apply the method to a simulated admixed dataset that was produced using real data and an evolutionary model previously used in [19]–[21]. In addition, we evaluate the performances of the algorithm on two real datasets, namely data from the HapMap project [22] and the Pan-Asian dataset [23]. A comparison of our method SHIPS and some of the main approaches that are Structure, Admixture, AWclust, and PCAclust is also conducted on these datasets.

## Methods

We present in this part the strategy of the SHIPS algorithm along with details of each step of the program. We also provide details about the methodologies of the other algorithms compared to SHIPS and the process used to assess all the methods. The simulated and real datasets analyzed are then described.

### The SHIPS algorithm

**Figure 1:**
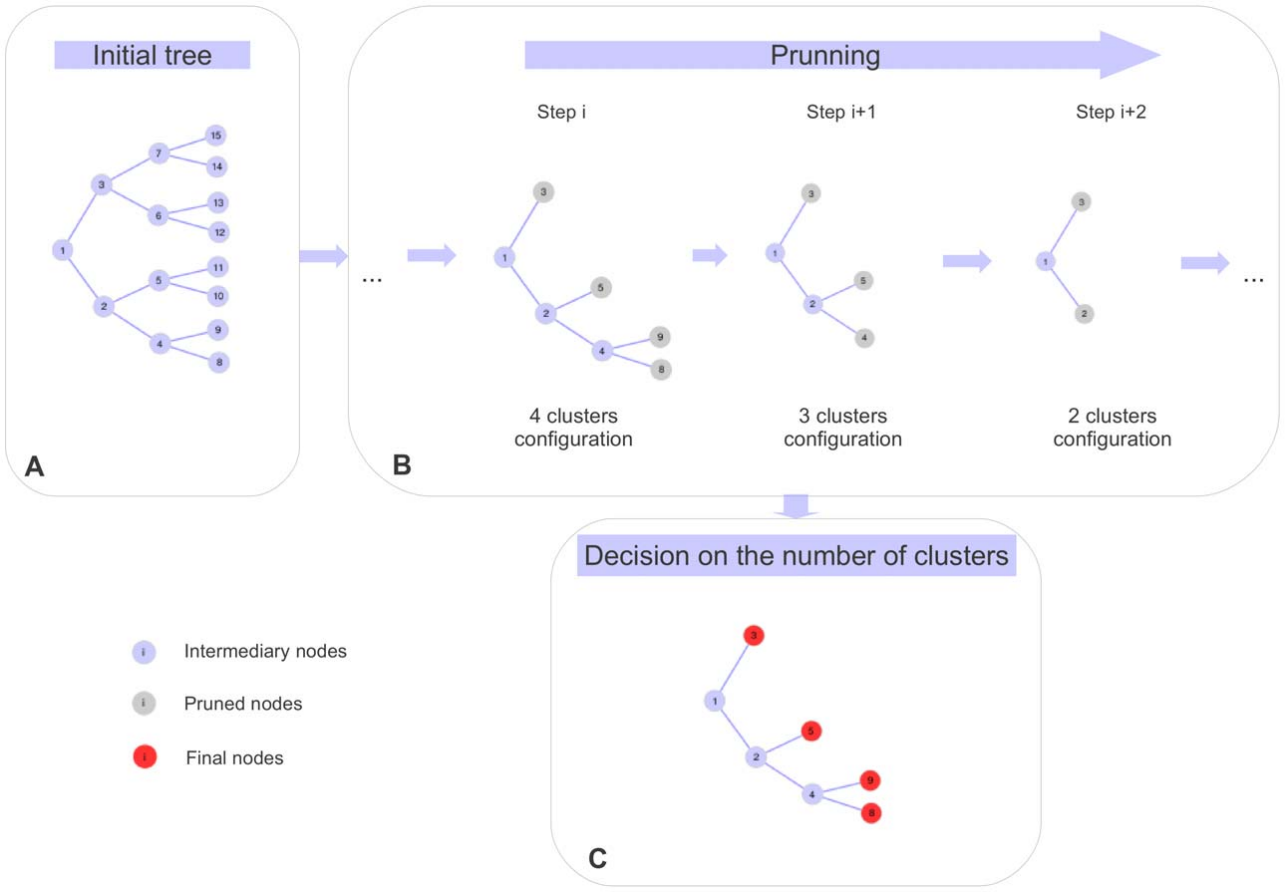
Graphical example of the SHIPS algorithm. After that the initial binary tree is built, the pruning procedure leads at the end of each step to a possible clustering of the individuals. In this example the data is clustered in four, then three then two clusters (gray nodes) at step *i*, 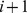 and 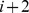 respectively. The final clusters decided by the gap statistic correspond to the ones of the four classes clustering (red nodes).

SHIPS can be described in several steps that are graphically represented in Figure 1.

1. Computation of a distance matrix that is a similarity matrix *S* between each pair of individuals. This matrix is used for the next steps of the algorithm.
2. Creation of a binary tree. Each population is divided in two sub-populations and so on (Figure 1-A).
3. Pruning of the tree to keep only the relevant branches corresponding to the relevant divisions (Figure 1-B).
4. Estimation of the optimal number of clusters *K* to determine which clusters of the tree are the final ones (Figure 1-C).

#### Similarity matrix

SHIPS is based on a spectral clustering algorithm. A similarity matrix is therefore necessary to apply this clustering method. We decided to consider a similarity matrix based on the allele sharing distance (ASD) that has been previously used to identify genetic patterns among populations [4], [17]. This matrix represents how close the genomes of each pair of individuals are. The similarity at SNP *l* between samples *i* and *j* is calculated as follows
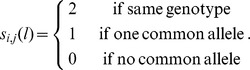



The total similarity between samples *i* and *j* is
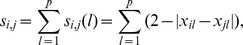
where 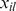, 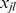 are the sample genotypes coded 0, 1 or 2 according to the number of reference alleles present at the locus *l*. The final matrix *S* = 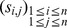 is a squared matrix of dimension 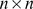, *n* being the number of individuals.

One has to note that any pairwise similarity matrix could be used in the algorithm instead of the one presented here. Examples of such matrices, based for instance on haplotypes instead of genotypes, are presented in [24]–[27]. We decided the choice of this similarity measure as it is fast to compute and led to high empirical performances of the algorithm.

#### Creation of a binary tree with successive spectral clustering algorithms

The binary tree produced by SHIPS is obtained by successively dividing each population in two sub-populations using a spectral clustering algorithm. Spectral clustering methods cluster points using eigenvectors of matrices derived from the initial data. We decided to use the version of this method proposed by Ng et al. [28], [29] that is the normalized spectral clustering described in the three following steps.

First, the similarity matrix *S* computed in the previous section is transformed into its normalized laplacian 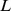 with
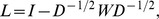
where 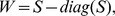 *I* is the identity matrix and *D* is a diagonal degree matrix such as each diagonal term 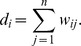
In a second step, a singular vector decomposition of the laplacian 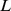 = 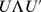 is computed and the 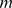 first eigenvectors (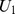, 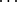, 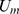) are normalized to get new vectors (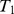, 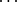, 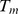), with norms of 1, defined by
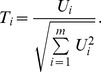
These vectors are used to cluster the points, i.e. divide a population in two sub-populations. Note that 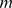 represents here the number of desired clusters so 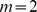 in the case of the SHIPS algorithm.

In a third step, a clustering algorithm is applied to the new vectors 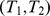 to create the two sub-populations. We decided to use a Gaussian mixture model (GMM) clustering after determining empirically that the usual *k-means* clustering algorithm is less robust than the GMM clustering when applied to our genetic data. The GMM clustering is used in the way the *k-means* would be, that is by strictly fixing the number of estimated clusters to 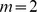.

If the population that we wish to split in two sub-populations is deemed homogeneous by the algorithm, the GMM clustering creates two clusters, one with all the samples and an empty one. This is a termination criterion that defines the end of a branch of the tree, called a terminal node. In extreme cases, the terminal nodes are all composed of a unique sample of the original population which ensures the convergence of the tree building step of the algorithm.

#### Pruning of the tree

The divisive strategy of SHIPS consists in dividing the original population in two sub-populations with the spectral clustering algorithm previously described and to iterate this procedure within each sub-population. This process leads to the computation of a binary tree (Figure 1-A). It is however noticeable that certain divisions are not relevant enough in terms of separating really distinct genetic populations. As a result, a pruning procedure is applied to the tree to progressively suppress the nodes, and the corresponding branches, that are the less relevant. This procedure creates several nested trees, each corresponding to a possible clustering of the individuals with a decreasing number of clusters (Figure 1-B). At the last step of the pruning, all the samples are in the same cluster.

The strategy of tree pruning that we use is the reduced error pruning. A quality indicator is defined and calculated for each node of the tree. This indicator is based on the sum of the squared similarities of a node and of its leaves. We define the function calculating the sum of squared similarities within a node 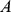 by
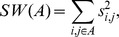
where 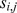 is the similarity previously introduced between samples *i* and *j*.

Considering a tree 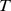, the quality of a node 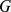 which has the leaves 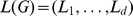 is defined by
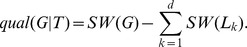
In terms of inter-cluster sums the quality can be expressed by
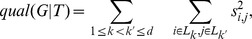
which corresponds to the sum of squared similarities between the leaves of *G*.

At each step, the node with the lowest quality value, 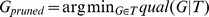, is pruned along with the subtree which it is the root. The indicators are recalculated after each step to account for the new topology of the tree.

#### Estimation of the optimal number of clusters

**Principle.** The optimal number of clusters *K* is regarded as a variable that is estimated using Tibshirani et al.'s gap statistic [30]. This method compares a quality indicator calculated on the result of a clustering in *k* classes of a dataset of interest and the value that this indicator would take under its null distribution, that is when the same clustering algorithm is applied to cluster a null reference dataset in *k* classes also.

A range of possible numbers of clusters, 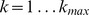, is thus investigated and for each an indicator 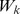 is calculated. The gap statistic is defined for a clustering with *k* clusters by
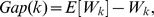
and estimated by
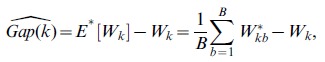
where 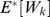 represents the expectation from the null distribution and therefore the 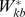 are the quality indicators calculated on *B* simulated null reference datasets. The simulation process for these datasets is described hereafter.

Several possible estimations of the optimal number of clusters *K* exist. We use the one proposed by Dutoit et al. [31] that is 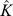, the smallest *k* such as
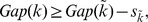
where 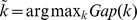 and 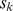 = 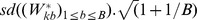. Note that the factor 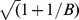 accounts for the simulation error of the 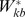.

#### Quality indicator

Let 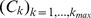 be possible clusterings of the samples in the data with *k* clusters in a clustering 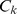. These clusterings are in our algorithm the ones determined at each step of the pruning (Figure 1-B). We call 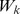 the quality indicator calculated on the clustering 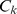. If we denote 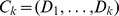, where 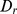 is the 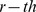 cluster of 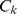, then the indicator that we consider is
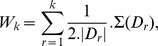
where 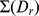 is the sum of the squared dissimilarities between the samples of the 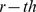 cluster of 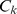 and 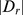 its cardinal (i.e. the number of samples in 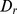). The dissimilarities are calculated like the similarities by inverting the values (0 if the samples have the same genotypes and 2 if they have no common alleles.)

In the classical version of the gap statistic, the logarithm of 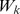 is used however several alternatives have recently been investigated [32]. We decided to use the aforementioned criterion as we observed that it led to a better estimation of the number of clusters for both our simulated and real genetic data.

#### Simulation under the null distribution

To simulate null reference datasets we simulate datasets with a number of variables and individuals identical to the one of the original datasets. Each variable was taken uniformly within 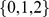 to match the SNPs values of the original datasets. Simulated that way, the null datasets correspond to data where there is no structure of the population. This simulation choice is also the one made in the algorithm AWclust that uses a gap statistic method. Note that theoretically it is not necessary to match all of the features of the data, such as for example the minor allele frequency of each SNP, when simulating under the null. This choice of simulation model was motivated by the empirical performances of the corresponding gap statistic to estimate accurate numbers of clusters in our applications.

#### Adequacy of SHIPS and the gap statistic

SHIPS has the advantage of producing in one run of the algorithm nested clusterings of the samples for 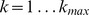 which renders faster the computation of the gap statistic. Note also that the quality indicator used in the gap statistic is based on a dissimilarity matrix while SHIPS uses a similarity matrix. This actually does not imply the computation of a new matrix, as the dissimilarity and the similarity matrix are linearly related. The gap statistic is therefore well suited to determine the optimal number of clusters with this new method.

#### Implementation

The SHIPS algorithm was implemented in R (http://cran.r-project.org) and the Mclust package was used within the spectral clustering steps to apply Gaussian mixture model clustering. A R package is freely available at http://stat.genopole.cnrs.fr/logiciels/SHIPS.

This algorithm takes as input parameters a SNP matrix of dimension 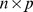 where *n* is the number of individuals and *p* the number of SNPs. Each entry of the matrix is coded 0, 1 or 2 given the number of reference alleles present at each locus for each sample. It is also necessary to indicate the maximum number of clusters to be investigated (denoted here 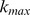) and the number of null datasets simulated (*B* here) to apply the gap statistic. A default value of 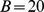 is set in the program.

### Evaluation of the method

A comparison study was conducted to assess the potential of SHIPS. Both simulated and real genotype datasets were considered and a panel of other methods was also applied to these data to conduct a comparison of their performances.

#### Methods included in the comparison

We compared SHIPS to some of the most commonly used clustering algorithms in the genetic field. We first considered the parametric approaches Structure and Admixture. Also we included a non-parametric approach, namely AWclust, and finally we added the alternative clustering strategy PCAclust to the comparison. We briefly describe the methods and the parameters used in this part and a detailed methodology of each of these algorithms is provided in Methods S1.

SHIPS was used with the default parameters, i.e. 20 null datasets simulated for the gap statistic. A reasonable maximum number of clusters was considered for all the methods, for instance, when analyzing a dataset with 10 (known) sub-populations we investigated up to 20 possible sub-populations.

Structure is a parametric algorithm that uses Bayesian statistical inference to cluster individuals. The version 2.3.2.1 was downloaded from http://pritch.bsd.uchicago.edu/structure.html and used with 5,000 burn-ins, 5,000 runs, the admixture model and no LD model. Structure provides a way of estimating the optimal number of clusters *K* through the model likelihood however it has been demonstrated that this method had shortcomings compared to more recent algorithms such as for instance Structurama [33] that allows a better estimation of *K*. To consider the best use of Structure, we therefore decided to opt for a way of estimating the number of clusters that advantages this method. In our comparison strategy a criterion is used to compare the different programs and we considered an estimated *K* for Structure that optimizes this criterion. Also, as Structure provides admixture proportions under the admixture model, we decided as it is usually done that an individual was assigned to the estimated population it has the highest probability to belong. Note that with this assignment method, certain clusters computed by the admixture model might not have any individuals assigned to them. In such a situation we considered the estimated number of clusters to be the effective number of sub-populations after the assignment procedure.

Admixture is also a parametric method that similarly to Structure model the ancestry proportions. It is based on the same statistical model but the optimization of the likelihood is enhanced. The program was downloaded from http://www.genetics.ucla.edu/software/admixture/download.html. The estimation of the number of clusters was conducted using the minimum of cross-validation error with the default parameter of 5 fold cross-validation. Like with Structure, we obtained discrete clusterings with this program by assigning an individual to the population it has the highest probability to belong.

AWclust uses a hierarchical clustering. The version 2.0 was downloaded from http://AWclust.sourceforge.net/ and used with the default parameters and 20 simulations for the computation of the gap statistic. The estimated number of clusters was determined using the maximum of the gap statistic.

PCAclust consists in computing a principal component analysis of the genotype data and then to apply a clustering algorithm, namely a Gaussian mixture model clustering, to the principal components such as described in [6]. The PCA was conducted using the software Eigensoft 3.0 developed by Patterson et al. [34], [35] and downloaded from http://genepath.med.harvard.edu/reich/Software.htm. The R package Mclust was used to apply GMM clustering to the set of relevant principal components selected with the use of the Tracy-Widom statistic. The optimal number of clusters was estimated using the likelihood computed by Mclust.

#### Population structure scenarios

We assessed SHIPS and the other methods on several datasets. We considered simulated datasets where the structures of the populations were controlled, a simulated admixed dataset and real datasets to determine the performances of the different approaches in real situations. For all of these scenarios small datasets of thousands of markers and large datasets of hundreds of thousands of markers were considered. We used several replicates for the small data in order to account for the simulation process or the markers sampling. Only one was used for the large scenarios due to the computational cost of certain algorithms.

#### Simulated datasets

**Figure 2:**
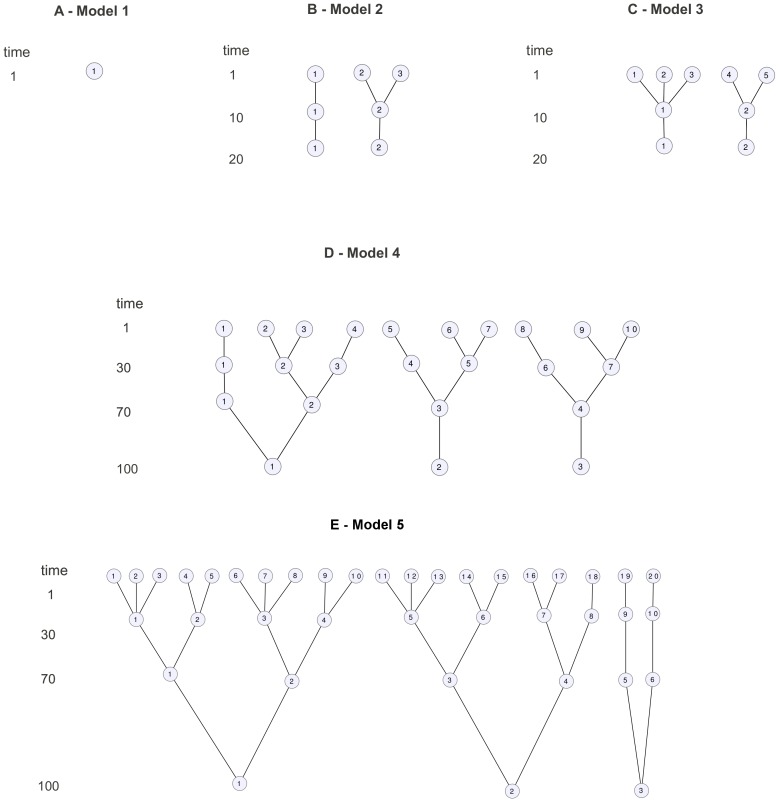
Population history trees used to generate the simulated datasets. A) one population B) three sub-populations C) five sub-populations D) ten sub-populations E) twenty sub-populations.

We simulated datasets using the software Genome based on the coalescent approach. We considered a first model M1 with no structure of the population in order to determine which methods are capable of uncovering that the data is not structured. We then considered 4 structured models, M3, M5, M10 and M20 with respectively 3, 5, 10 and 20 sub-populations and increasing complexities of population histories. Figure 2 presents the population histories of these models and table S1 the detail of the sampling. The models used in Genome for the simulations are provided in Methods S2. Each small dataset is composed of 5,000 SNPs and each large dataset of 200 K SNPs simulated in equal number on each of the non-sexual chromosomes. Ten datasets were simulated and analyzed by the algorithms for each small scenario. The results are then averaged over these datasets. Note also that for computational purposes, Structure was only applied to five small datasets and was not applied to the large ones.

#### Simulated admixed datasets

In order to assess the performances of the various algorithms on more realistic situations we simulated a discrete admixed dataset corresponding to the model named Madx. Two real populations from the HapMap phase 3 data, namely the Han Chinese from China (CHB) and the Utah residents with Northern and Western European ancestry from the CEPH collection (CEU), were used in an evolutionary model to produce an admixed population. The evolutionary model consists in randomly mating samples from each of the two original populations and to iterate this process over time. The final dataset is composed of the two original populations (CEU and CHB) and the admixed simulated one (named XY). The detail of the sampling is provided in Table S2. Like for the other simulated datasets we considered small data of 5,000 SNPs with ten replicates and one large data of 200 K SNPs.

#### HapMap dataset

**Figure 3:**
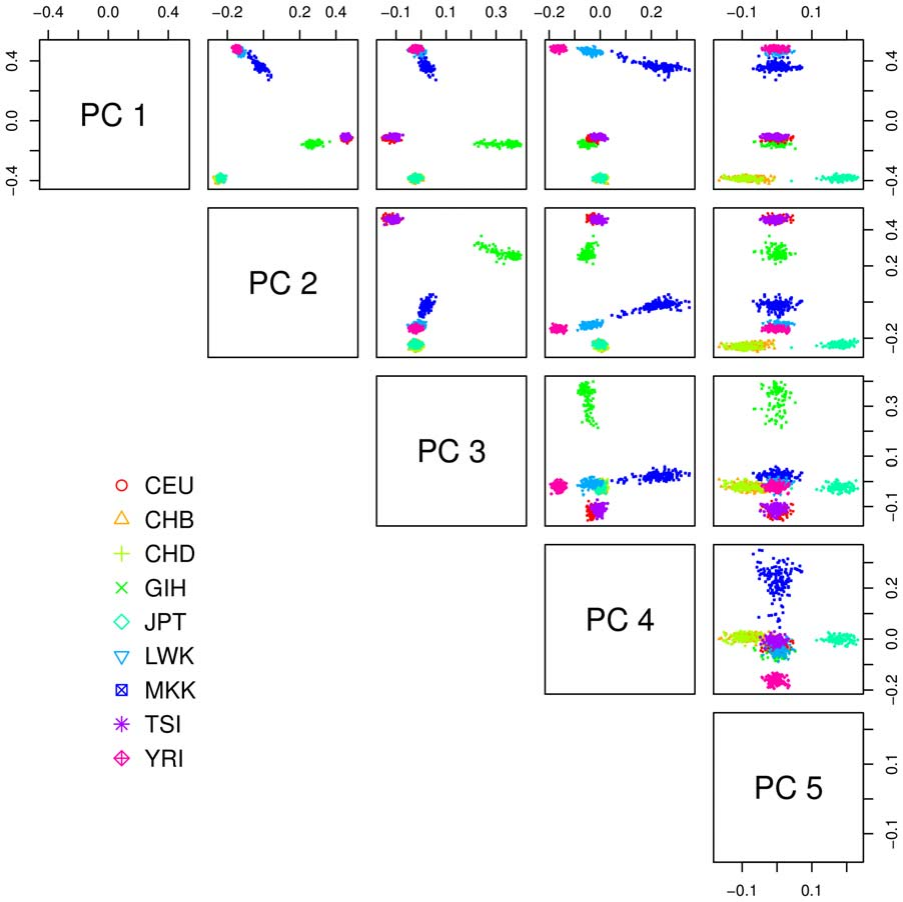
Representation of the 9 populations of the HapMap dataset. This scatter-plot uses the first five principal components of a dataset with 20 K SNPs. This graph is only intended to present the general genetic pattern of the dataset and does not exhaustively represent the capability of the PCA to separate the populations.

We also focused on the potential of the methods when applied to real datasets. We first considered the HapMap phase 3 dataset with 9 populations and 1,087 individuals (Table S3). Figure 3 is a graphical representation of the populations on the principal components space. We considered small data with 20,000 SNPs and large data with 220 K SNPs randomly chosen among the whole set of SNPs available and in equal number on each of the non-sexual chromosomes. To account for the SNPs sampling, twenty replicates of the small HapMap data were considered to assess the methods, except for Structure that was only applied to five datasets. The HapMap dataset is available at http://hapmap.ncbi.nlm.nih.gov/downloads.

#### Pan-Asian dataset

**Figure 4:**
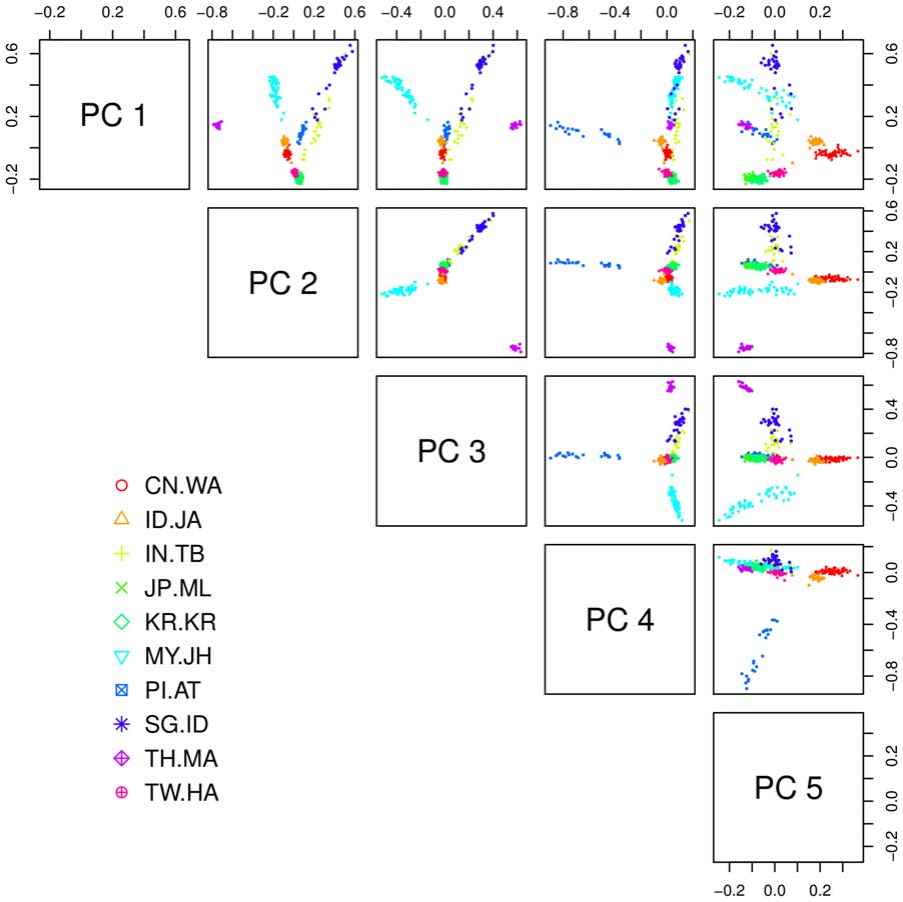
Representation of the 10 populations of the Pan-Asian dataset. This scatter-plot uses the first five principal components of a dataset with 20 K SNPs. This graph is only intended to present the general genetic pattern of the dataset and does not exhaustively represent the capability of the PCA to separate the populations.

The PASNPi consortium provides the genotype data of 75 Pan-Asian and HapMap populations with 1928 individuals and 54,794 SNPs. Among all these populations, certain main groups, defined by the countries of origin, can be highlighted. We focused on 10 sub-populations formed by 443 individuals, from each of these groups (Table S4, Figure 4) and refer to these data as the Pan-Asian datasets. Like for the HapMap data, we selected 20,000 SNPs randomly chosen in equal number on each of the non-sexual chromosomes among the initial dataset for the small data (with twenty replicates) and the whole set of SNPs for the large data. For the reasons indicated previously, Structure was only applied to five small replicates. The complete PANSNPi dataset is available at http://www4a.biotec.or.th/PASNP/

#### Assessing the clustering quality

To assess the potential of a clustering method it is important to focus on both the sample assignments and the estimated number of clusters. The quality indicator usually considered is the accuracy, that is the proportion of individuals that were assigned to the correct populations. This indicator focuses only on the one-to-one relationship between estimated clusters and true populations. We decided not to retain this criterion as it does not exhaustively describe the quality of a clustering method's assignments and does not account correctly for the estimated number of clusters. The indicator we selected to account for both the assignments and the estimation of the number of clusters is the adjusted Rand index [36]. This index is calculated using the contingency table of two clusterings *U* and *V* (Table 1) with the formula
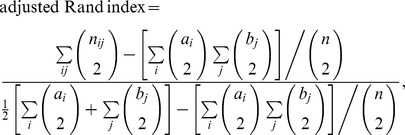
where 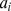 and 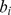 are the numbers of samples in the *i-th* clusters of *U* and *V* respectively and 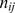 the number of samples in the *i-th* cluster of *U* and the 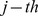 cluster of *V*.

**Table 1:** Contingency table between two clustering *U* and *V*.

	*V*_1_	*V*_2_	…	*V*_c_	*Sums*
*U*_1_	*n*_11_	*n*_12_	…	*n*_1c_	*a*_1_
*U*_2_	*n*_21_	*n*_22_	…	*n*_2c_	*a*_2_
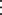	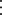	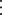	<$>\raster(60%)="rg2"<$>	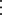	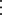
*U_R_*	*n_R_*_1_	*n*_R2_	…	*n_RC_*	*a*_R_
*Sums*	*b*_1_	*b*_2_	…	*b_c_*	*N*

*a*_i_ and *b*_i_ are the numbers of samples in the *i-th* clusters *U_i_* of *U* and *V*_i_ of *V* respectively and *n*_ij_ the number of samples in the *i-th* cluster *U_i_* of *U* and the *j-th* cluster *V*_j_ of *V*.

This index focuses on all pairs of samples and considers whether they have correctly been assigned to the same population or correctly been assigned to different populations. That way, in addition to the accuracy criterion, the adjusted Rand index takes into account the fact that certain samples should not be clustered together. The adjusted Rand index is comprised between 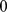 and 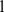, a value of 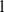 meaning a perfect clustering. Note that if there is only one cluster in the data and that a clustering method properly uncovers such a structure the Rand index is theoretically not defined. Given that the structure is perfectly estimated in such a case we consider a value of 1 for the Rand index.

For simulated datasets we compared, via the adjusted Rand index, the clusterings proposed by the different methods to the true population labels that are available through the simulation process. For the admixed and the real datasets, no true population labels exist. As a consequence we provide two quality measures that are the quality index using as comparison partitions the population labels provided with the datasets (e.g CHB or CHD in HapMap) and the partitions produced by Admixture. We selected Admixture as it is one of the most widely used methods for the estimation of population structure. Also we represent the admixture proportions of all the methods with barplots. For discrete clusterings these proportions are either 0 or 1.

## Results

**Figure 5:**
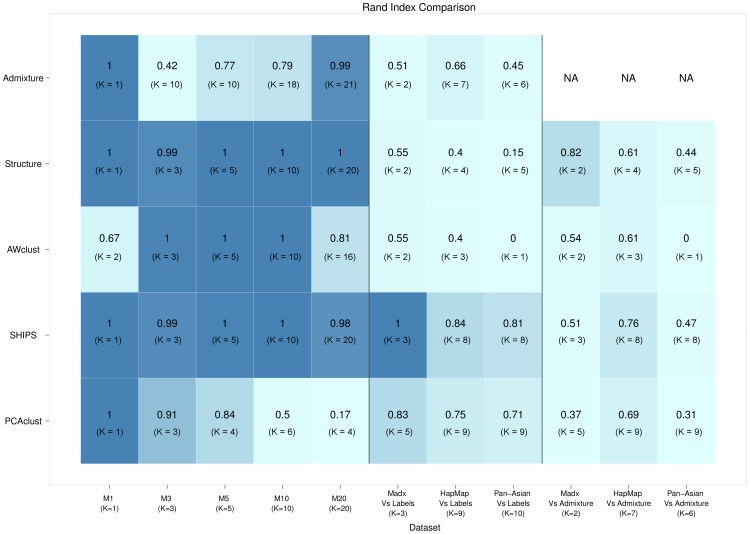
Comparison of the clustering methods on the small datasets. Average Rand indexes over all small replicates are indicated for each method and each model along with the estimated number of clusters in parenthesis. The darker a cell color is, the better the corresponding clustering is.

**Figure 6:**
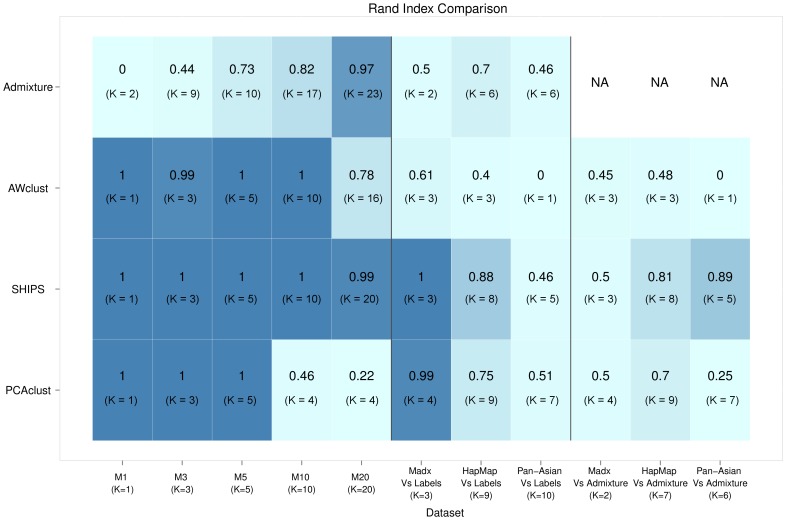
Comparison of the clustering methods on the large datasets. Rand indexes are indicated for each method and each model along with the estimated number of clusters in parenthesis. The darker a cell color is, the better the corresponding clustering is. the software Structure was not applied to large datasets due to a too large computational cost.

Several small datasets and one large dataset were investigated for each simulated or real scenario. The average Rand indexes and the average estimated numbers of clusters are the indicators we are interested in. Figure 5 presents these values for all the methods applied to small datasets and Figure 6 for the large datasets. In addition, Figures S1, S2, S3, S4, S5, S6, S7, S8 provide examples of the graphical representations of the criterion used by SHIPS to estimate the number of clusters *K* and Table S5 the average numbers of principal components retained by the algorithm PCAclust in each scenario.

### Simulated datasets

Figures S9, S10, S11, S12 provide graphical results of SHIPS applied to the small simulated datasets and Figures S13, S14, S15, S16 when applied to the large ones.

#### Model M1 (1 sub-population)

For the model M1, with only one population, SHIPS was always able to correctly determine the correct number of one cluster for both all the small and large datasets. This was also the case of Structure and PCAclust. As a consequence these three methods perfectly assigned all the individuals to the correct population and had a Rand index of 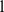. On the other hand, Admixture was only able to determine that there was no structure in the small datasets, estimating 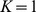, but not in a large dataset producing 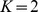. This is bound to be due to the number of SNPs that led the algorithm to determine a more complicated structure. AWclust properly determined that there was one cluster in 7 small replicates out of 10, but the average number of estimated clusters is 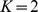. On the large dataset, this latter method correctly estimated the number of clusters as the amount of SNPs allowed the AWclust's gap statistic to be more accurate.

#### Model M3 (3 sub-populations) and M5 (5 sub-populations)

The performances of SHIPS, Structure and AWclust were comparable for the models M3 and M5. An average number of 3 and 5 clusters was respectively estimated for all small and large replicates of the models M3 and M5 (except for Structure that was not applied to large datasets). These three methods mis-classified in average less than 3 individuals leading to Rand indexes higher than 0.99. PCAclust was able to estimate the correct number of 3 sub-populations in 8 small replicates out of 10 small datasets of the model M3 and in 5 replicates for the model M5. When the number of SNPs increased to 200 K, PCAclust was able to correctly estimate *K* and led to perfect sample assignments. The clustering proposed by Admixture on these models were not consistent with the true populations. Indeed, this method identified the maximum number of clusters to be the optimal one, that is 10 in our case. Larger sample size did not improve these results.

#### Model M10 (10 sub-populations)

The model M10, with 10 populations, pertains to a more complex structure of the data. In this scenario SHIPS, Structure and AWclust succeeded in perfectly estimating *K* and assigning all individuals to the correct populations for both small and large datasets. PCAclust estimated a mean number of 6 clusters for the small data, 4 for the large data as it was not able to separate certain populations. Admixture again over-estimated the number of clusters (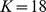 for small data and 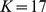 for large data). We investigated up to 20 clusters but the algorithm did not converged for values of *K* greater than those estimated.

#### Model M20 (20 sub-populations)

In this last simulated model, with the more complex structure and 20 populations, both SHIPS and Structure evaluated the correct number of clusters for all replicates and completed an individual assignment very consistent with the true populations. AWclust and PCAclust underestimated the number of clusters. AWclust only allows to estimate a maximum of 16 clusters that was reached for this complex dataset. One could wonder if the clustering assignments would have been better if the maximum number of clusters was more flexible. On the other hand, PCAclust was not able to detect the structure of this dataset. Only 4 clusters in average were identified in the small and large datasets as many populations were not separated thus leading to a low Rand index close to 0.2. For both small and large datasets Admixture estimated 21 clusters and almost perfectly assigned all the individuals to the correct populations. Even though these clusterings are quite accurate, it is noticeable that 21 was the maximum number of clusters for which the algorithm converged. In other words, it is possible that if the convergence could have been reached for greater values of *K*, the number of clusters could have been over-estimated again.

SHIPS and Structure were the most accurate methods when applied to simulated datasets both in terms of estimating the correct number of clusters *K* and assigning individuals consistently with the true population labels. The performances of the other methods were a little less, especially for Admixture that always over-estimated *K* and PCAclust that usually under-estimated it. It is also noticeable that for all of the methods the results are generally comparable between the large and the small datasets.

### Admixed and real datasets

**Figure 7:**
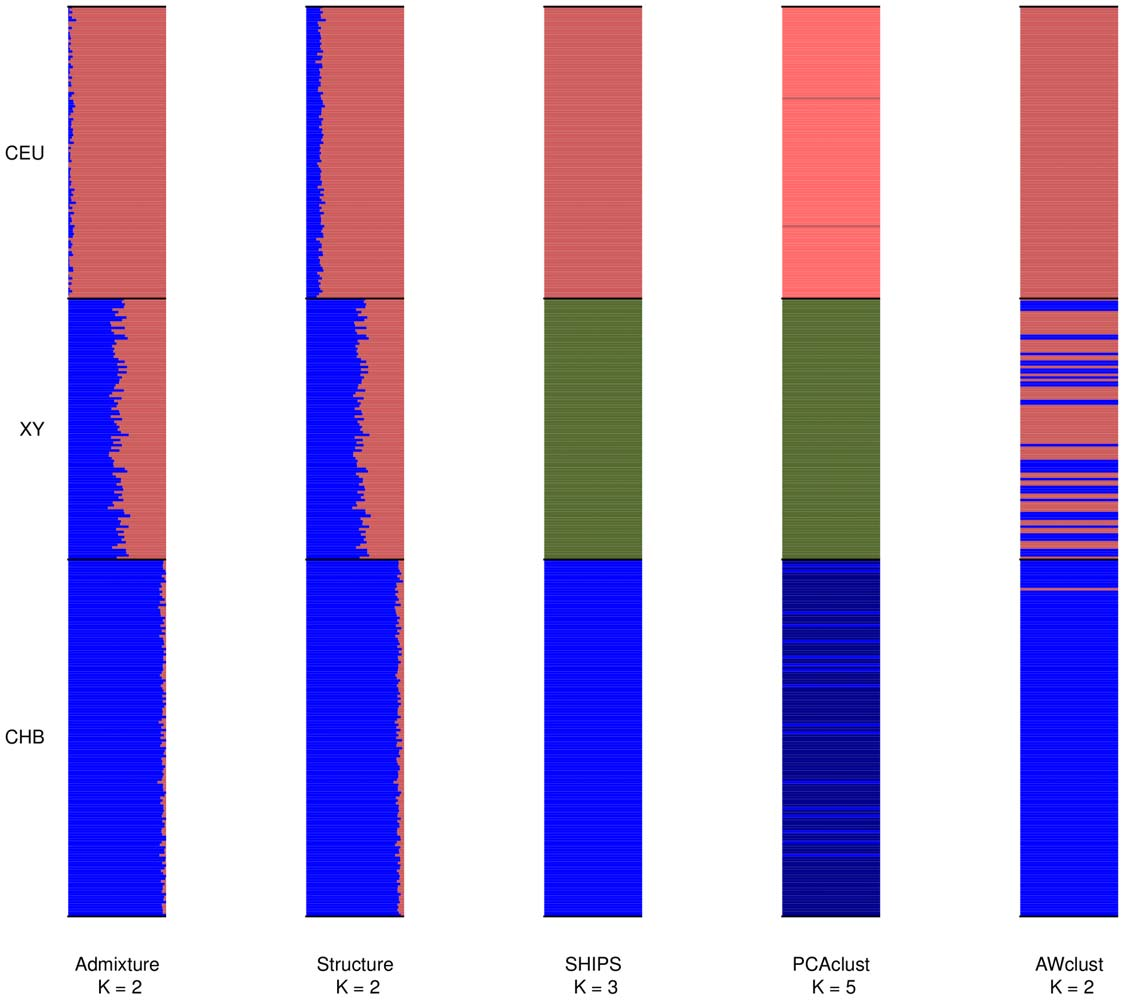
Barplots of the admixture proportions for the small admixed data. The first small dataset was used to produce this plot. Populations are separated by black lines and assigned with a unique color that is approximatively reported on the barplot of each method. For the discrete methods the admixture proportions are either 0 or 1.

**Figure 8:**
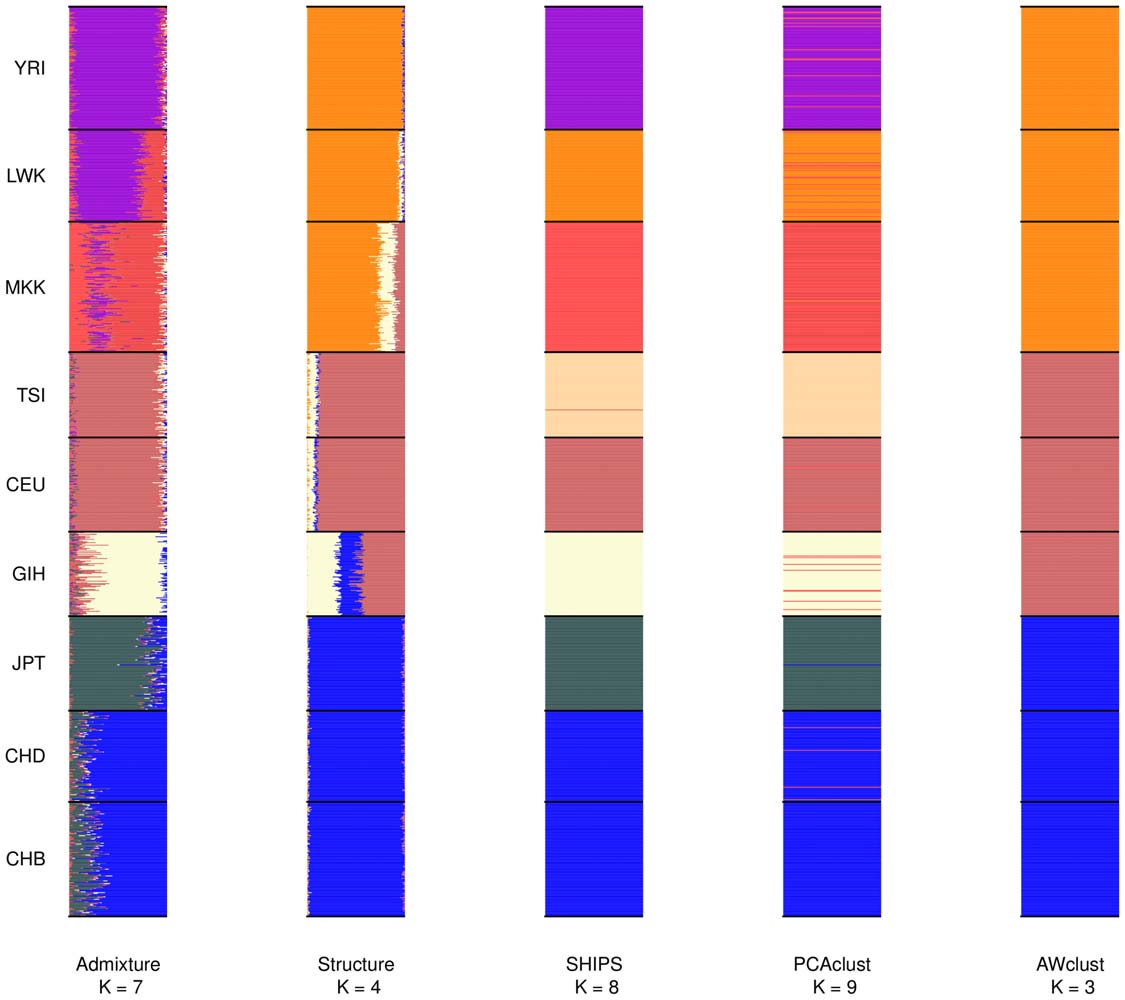
Barplots of the admixture proportions for the small HapMap data. The first small dataset was used to produce this plot. Populations are separated by black lines and assigned with a unique color that is approximatively reported on the barplot of each method. For the discrete methods the admixture proportions are either 0 or 1.

**Figure 9:**
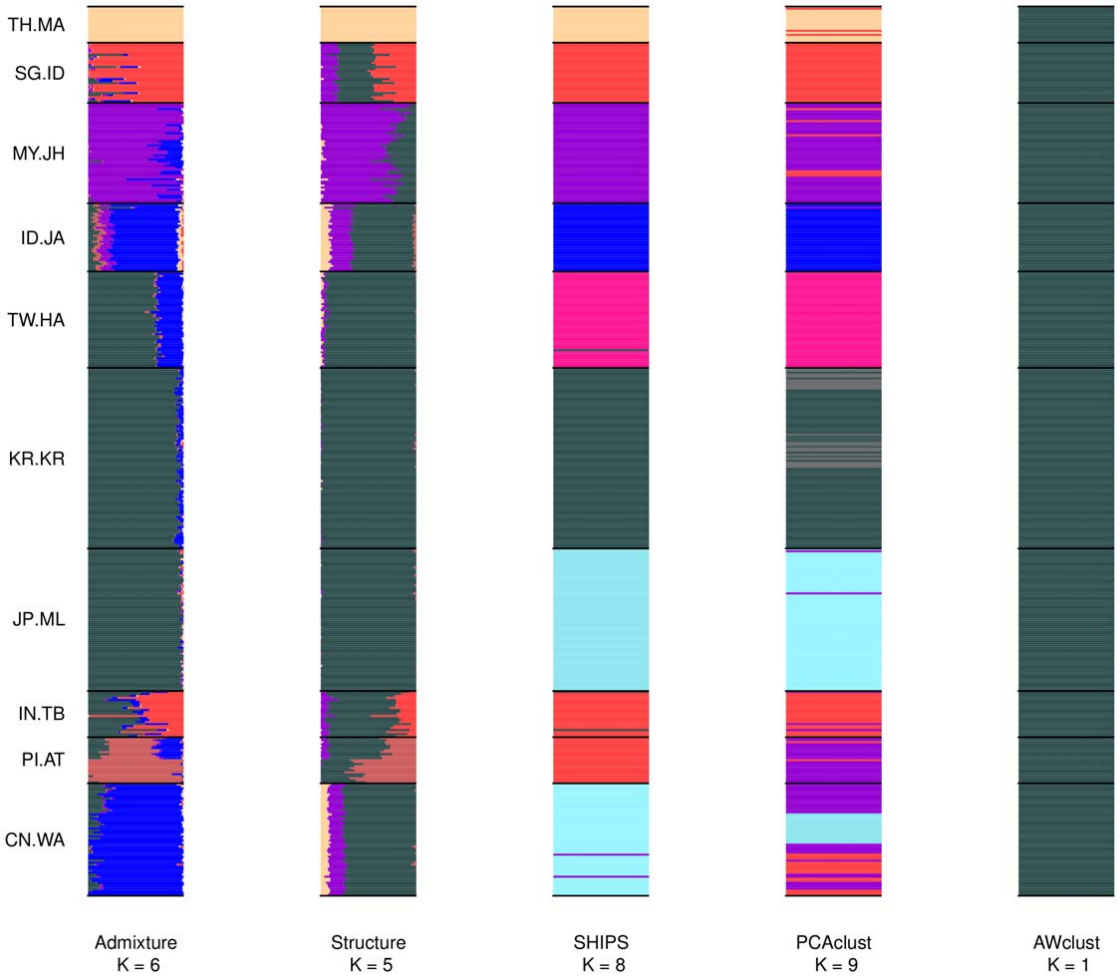
Barplots of the admixture proportions for the small Pan-Asian data. The first small dataset was used to produce this plot. Populations are separated by black lines and assigned with a unique color that is approximatively reported on the barplot of each method. For the discrete methods the admixture proportions are either 0 or 1.

**Figure 10:**
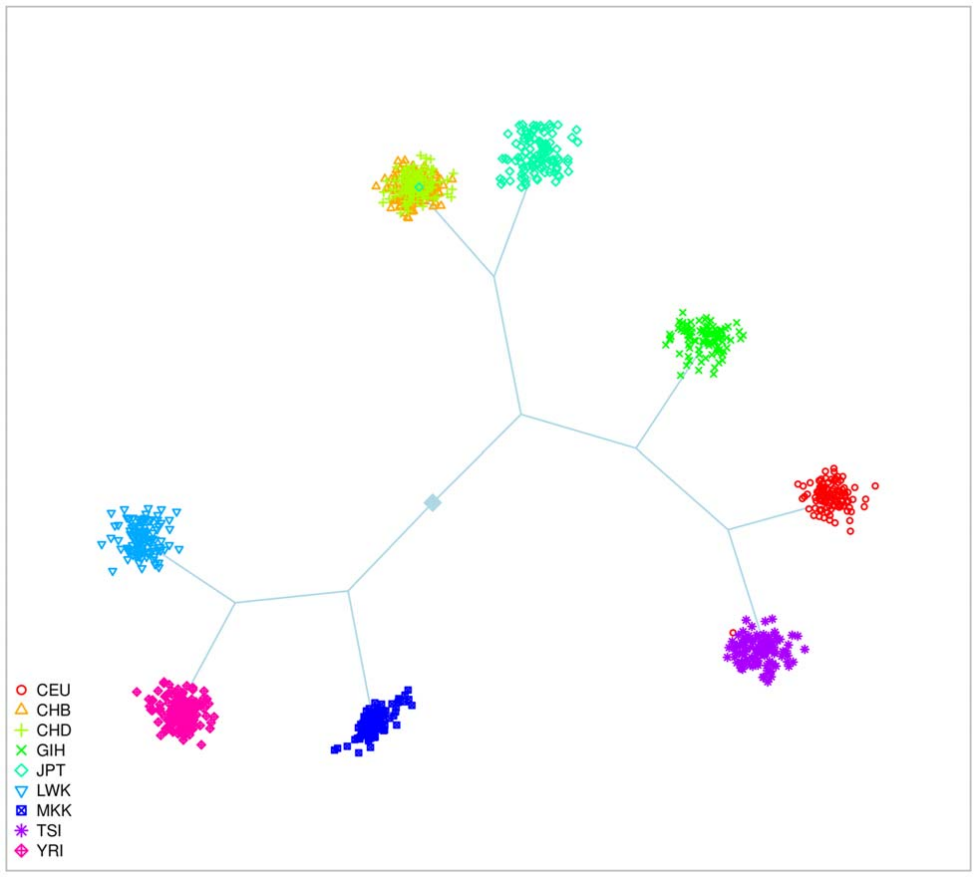
SHIPS tree of the 9 HapMap populations. This representation is an output produced by SHIPS. The tree structure corresponds to the successive divisions conducted by the algorithm. Each final cluster is represented by a scatter-plot of its members. We colored here the individuals according to the population labels.

**Figure 11:**
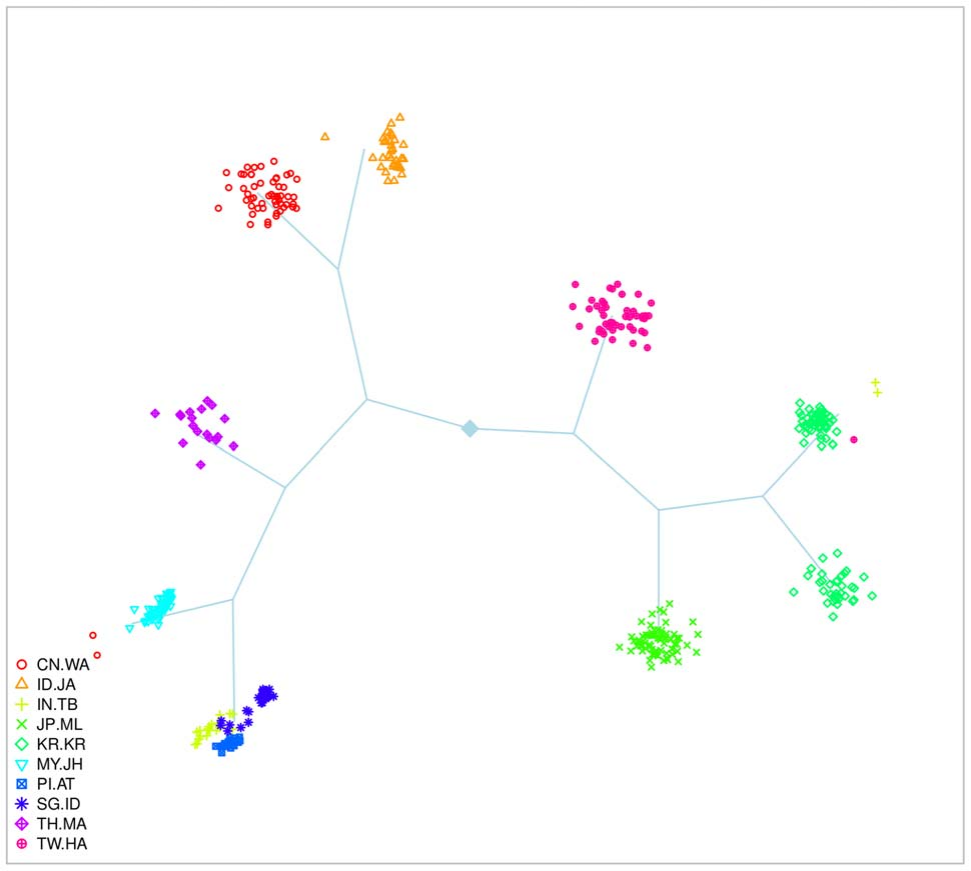
SHIPS tree of the 10 Pan-Asian populations. This representation is an output produced by SHIPS. The tree structure corresponds to the successive divisions conducted by the algorithm. Each final cluster is represented by a scatter-plot of its members. We colored here the individuals according to the population labels.

In order to assess the quality of the clustering methods we were also interested in looking at admixed and real datasets, more representative of the ones encountered in genetic studies. We present the average results over the different small and large replicates, along with details on the assignments performed. In order to account for the fact that there is no “true” structure in real datasets, we considered both the population labels and the labels produced by the program Admixture as structures (also called partitions) of reference. Figures 7, 8, 9 are the barplots of the admixture proportions of the different methods for the small datasets and Figures S19, S20, S21 are the same representations for the large datasets. In addition Figures 10 and 11 display the graphical results of SHIPS for the small HapMap and Pan-Asian data and Figures S17 and S18 the counterpart for the large datasets.

#### An admixed population

SHIPS identified 3 distinct populations for the admixed datasets that are the two populations of origin (CEU and CHB) and the one simulated as an admixture. Structure, Admixture and AWclust detected two populations. The admixture proportions displayed in Figure 7 show that Admixture and Structure estimated almost the same ancestries for the individuals, with the admixed population (XY) having a genome coming approximately in equal part from the CHB and CEU populations. These proportions correctly match those used in our simulation model. AWclust resulted in a split of the admixed population in function of these admixture proportions. On the other hand, PCAclust estimated 5 clusters that correspond to the 3 distinct populations identified by SHIPS and two small clusters being sub-populations of the CHB and CEU populations.

In terms of quality indexes, when comparing to the population labels, SHIPS and PCAclust performed the best as they identified the 3 main discrete populations. When comparing the results to Admixture, Structure is the closest in such a setting and SHIPS and AWclust are in agreement at about 50% as they assigned the samples from the admixed population to another population being a cluster of admixed, CEU or CHB individuals.

The results are quite similar on the large admixed dataset except PCAclust that did not find small sub-clusters within the CHB populations (Figure S19).

It is interesting to notice that there are two kinds of behaviors to cluster the admixed individuals. Certain methods assigned them to the populations of origin they are the closest genetically speaking and others created a specific admixed cluster. These two behaviors of the methods are understandable given the nature of the admixture that we considered in this simulation. Indeed, we simulated a discrete admixture, meaning that the admixed samples, even though originating from the CHB and CEU populations, form a discrete cluster. The nature of this structure is therefore more challenging for discrete clustering algorithms such as SHIPS and AWclust but also quite favorable to discrete assignments compared to ‘real life’ admixtures that are usually continuous. The results produced by Structure and Admixture have to be interpreted in the sense that with a continuous admixture only the admixture proportions can properly relate the structure as there would be no discrete cluster to be identified. Further analyses of these algorithms on continuous admixture would reveal more precisely the behaviors of the algorithms with such population structure and complete the partial results presented here.

#### HapMap 9 populations

Considering all 20 small replicates, SHIPS was able to identify 8 clusters in average (Figure S7). Certain populations such as the two Chinese populations (CHD and CHB) were not entirely differentiated in some datasets. Also, two of the African populations YRI and LWK were sometimes assigned to the same cluster. Results were similar on the large dataset. In both cases, an average Rand index of about 0.8 was reached when using the population labels as reference (Figures 5 and 6). PCAclust estimated 9 clusters by assigning CHB and CHD to the same cluster and splitting certain populations such as GIH or the African ones into several clusters. Structure and AWclust produced clusterings less consistent with the population labels. Structure identified the three main ethnicities, that are African, Caucasian and Asian plus the GIH population. Note that this population derives from the Asian and Caucasian one. AWclust was only able to detect the three main ethnicities. These two latter methods have therefore relatively low Rand index (0.4) compared to the population labels.

Admixture estimated 7 ancestral populations in the small datasets. As we can observe on Figure 8, according to Admixture, the CHB and CHD populations share a very close ancestry, which can explain why SHIPS and the other methods did not split these populations. The JPT population has a common ancestry with the Chinese populations but with different admixture proportions. SHIPS and PCAclust were able to differentiate this population from CHB and CHD but not Structure and AWclust. Among the 7 ancestral populations detected by Admixture, one is specific to the GIH population. In addition, Structure uncovered the same admixture pattern which validates the clusterings of SHIPS and PCAclust that differentiated the GIH population. It is noticeable that even though the admixture proportions of the Caucasian population CEU and TSI are very close, SHIPS and PCAclust were able to separate them into two distinct clusters. The behavior of the methods is however different on the African populations. The 3 corresponding populations share the same 3 ancestries in different proportions. SHIPS differentiated these 3 populations correctly whereas PCAclust created a fourth cluster composed of samples from each of these populations. When observing the admixture proportions of the samples clustered into this additional group, there seems to be no common pattern and therefore this split appears to be inconsistent with the structure of the population. As a result SHIPS is the method that agrees the most with Admixture (Rand index = 0.76) followed by PCAclust (Rand index = 0.69), Structure (Rand index = 0.61) and AWclust (Rand index = 0.61).

On the large dataset, results are quite similar except that Admixture estimated 6 ancestral populations. The corresponding assignments were however more consistent with the population labels. The same observation can be made for SHIPS and as a consequence the quality indicator of our new method improved whether we compared it to the population labels or to Admixture.

#### Pan-Asian 10 populations

We first describe the results for the small datasets. In average, over all the small Pan-Asian datasets SHIPS estimated 8 clusters. In the majority of the replicates the population from India (IN.TB) was clustered with the Philippines (PI.AT) or Singapore (SG.ID) and the populations from China (CN.WA) and Indonesia (ID.JA) or Japan (JP.ML) were assigned to the same cluster. These clusterings of the data are quite consistent with the labels of the populations and as a consequence SHIPS has the highest Rand index of 0.81 with this reference partition. PCAclust estimated 9 clusters. The CN.WA population was split in several clusters and often assigned to the same clusters as samples from SG.ID and IN.TB or PI.AT and MY.JH. Several other populations were separated according to the population labels and therefore the quality index with this reference is of 0.71. Structure identified 5 ancestral populations. The corresponding discrete clustering is however quite distant from the population labels. Indeed, only the MY.JH, TH.MA and part of the SG.ID populations are separated. As a consequence the Rand index compared to the population labels is quite low. Likewise, AWclust has a null Rand index as this method did not determine any structure in the data. Admixture found 6 ancestral populations. The populations IN.TB, JP.ML, KR.KR and TW.HA were assigned to the same cluster like CN.WA and ID.JA. This results in a Rand index of 0.45. When analyzing the admixture proportions (Figure 9) we observe that SHIPS assigned the populations IN.TB and CI.AT to the same cluster whereas these populations share quite different ancestries. On the other hand, this novel algorithm differentiated the TW.HA, KR.KR and JP.ML populations that have closely related ancestries. PCAclust also assigned these populations to different clusters but had a lower Rand index than SHIPS compared to the Admixture partitions as the additional cluster detected by this method does not match the admixture proportions.

On the large datasets, SHIPS and PCAclust estimated fewer clusters than on the small datasets. SHIPS estimated 5 clusters and PCAclust 7 clusters. These differences resulted in SHIPS identifying a structure very close to that estimated by Admixture (Rand index of 0.89) while PCAclust's clustering was less in agreement with Admixture (Rand index of 0.25). On the other hand, PCAclust was closer to the population labels partition than SHIPS. One has to note that when setting the number of clusters manually, SHIPS and PCAclust estimated the same structure than on the small datasets. These different behaviors of the methods are therefore due to the size of the dataset that influenced the estimations of the number of clusters.

The analysis of the real datasets pointed out that compared to the population labels as reference partitions, SHIPS was the most efficient method to uncover the population structures followed by PCAclust. Even though SHIPS produces discrete clusterings, this novel algorithm reached the most important agreement with the clusterings estimated by widely used methods such as Admixture.

## Discussion

We have proposed in this paper a novel clustering approach to infer the genetic structure of populations from SNPs data. SHIPS is based on a divisive hierarchical clustering procedure and a pruning strategy followed by the use of the gap statistic to estimate the final number of clusters *K*.

SHIPS has proven to be an accurate and precise method to estimate both relevant optimal numbers of clusters as well as for producing assignments consistent with the reference partitions of the data considered. In the simulated datasets, *K* was always correctly estimated and only few individuals were mis-assigned. The structures identified for the admixed dataset (K = 3), the HapMap (K = 9) and the Pan-Asian (K = 10) datasets were remarkably close to the population labels or the partitions estimated by the program Admixture.

The other algorithms considered had less regular performances, either missing the structure of the complex simulated data or of the real datasets. A possible explanation of these results depends on the algorithms' methods to estimate the number of clusters or on the parameters utilized for each algorithm. It is interesting to observe that even though Structure and Admixture are based on the same model their performances are notably different. On the simulated datasets, Structure was able to estimate the correct *K* for each dataset. On the other hand, Admixture always over-estimated the number of clusters by selecting the higher *K* investigated. This led to poor performances of Admixture on the first simulated scenarios (M1 and M3) and relatively satisfying ones on the final scenarios (M5, M10 and M20) as the correct number of clusters corresponded to the maximum *K* for which the method converged and therefore the estimated *K*. Given that when manually setting *K* to the true values, Admixture identified the true structures of the data, the estimation of the number of clusters through cross-validation can be identified as the cause of the poor clustering quality of the algorithm on the simulated datasets. We considered different cross-validation methods that are 5, 10 and 15 fold cross-validation, and obtained the same estimations of *K* (data not shown). It therefore appears that the cross-validation method is not fit in such settings to estimate the number of clusters. These results confirm certain limitations of the cross-validation criterion that had already been pointed out [13], [26]. We used in our comparison an improved version of Structure by considering an estimated *K* maximizing the quality criterion thus leading to more correct estimation of *K*. However, one has to note that the estimation method originally used in Structure, that is the maximum likelihood, led to correctly identifying the structure of the simulated data (data not shown). The opposite conclusions can be drawn for real datasets (HapMap and Pan-Asian). Admixture estimated values of *K* close to the ones defined by the population labels while Structure under-estimated the values of *K* compared to both the population labels and Admixture. The cross-validation method used in Admixture is more appropriate for real complex datasets however there are no efficient way to estimate a correct *K* for Structure. This is due to the fact that even when setting manually *K*, Structure produced clusterings with empty clusters and therefore could not identified more populations than we presented in the Results section. For example, only the three main ethnicities plus the GIH population were identified in the HapMap data while other methods such as SHIPS or Admixture were able to differentiate the Asian, Caucasian or African populations. A possible explanation for Structure's results is that, even though the algorithm converged properly, a too short burn-in period and too few runs of the algorithm were used for such complex data. These choices were however made due to the very high computational time of the program.

AWclust generally uncovered the structure of the small and large simulated datasets but failed to properly analyze the real datasets. Whether we considered the population labels or the partitions produced by Admixture as reference for the real datasets, AWclust's clusterings were not in agreement with these references. Only the three main ethnicities were detected in the HapMap data and no structure in the Pan-Asian data due to the fact that the optimal estimated number of clusters were under-estimated. It is however interesting to notice that when manually setting the number of clusters, the sample assignments were more consistent with both the population labels or the results of Admixture. This can be explained by the gap statistic used by the algorithm that was not able to select the correct values of *K* while the hierarchical clustering could separate certain populations. 20 simulations for the gap statistics may not have been enough though the same number was used with SHIPS that more correctly estimated *K*. These results highlight the quality of the version of the gap statistic that we used in the SHIPS algorithm.

In addition to the individuals clustering, both SHIPS and AWclust provide tree structures that allow the analysis of the relationship between populations. The corresponding graphical representations, presented in Figures 10, 11 and S9, S10, S11, S12, S13, S14, S15, S16, S17, S18 for SHIPS, are quite similar to dendrograms produced by AWclust. The differences are that in SHIPS the lengths of the branches have no meaning and the individuals of the final clusters are plotted to represent their dispersion. The analysis of these two kinds of graphical representations were quite similar in our comparisons. For example, we observed in the simulated datasets, that for basic population structures (model M3 and M5), the trees provided by SHIPS and AWclust properly related the genetic histories of the populations. For more complex datasets, mainly the major population differentiations and some of the finer separations led to tree branches consistent with the population histories represented in Figure 2. Also, these representations can provide indications on the genetic distance of the real populations. For instance, we observed on Figure 10 that the Caucasian and Asian populations are first separated from the African ones and then separated from each other.

The method PCAclust selected the number of principal components to be used for the clustering using the Tracy-Widom statistic (Table S5). Many components (more than 25) were determined significant for the complex simulated datasets M10 and M20. This led to clusterings rather inaccurate as the estimated numbers of clusters were greatly under-estimated for both the small and large datasets. If fewer PCs were kept, e.g only five, the estimated *K* would have been more exact (data not shown). This indicates that too many PCs add a non-negligible noise to the data provided to the GMM clustering and therefore that the PCs selection method of PCAclust could be improved.

The performances of this method are however better when applied to real datasets, especially when compared to the population labels. When comparing the clusterings produced by PCAclust to Admixture, the results are more mitigated. PCAclust estimated more clusters than Admixture and split populations that this latter algorithm considered coming from the same ancestral populations. A reason might be that even though the two algorithms are somehow linked [27], the methods to estimate the numbers of clusters are quite different.

The methods discussed here are composed of two parts to analyze the structure of the populations. The first corresponds to the quality to assign individuals to relevant clusters and the other is the ability to estimate a proper optimal number of clusters *K*. If a potential value of *K* is unknown, it is important that the clustering method estimates a proper *K* otherwise even with accurate sample assignment capabilities the resulting clustering may not be relevant. Among all the algorithms that we investigated in this paper, SHIPS was the only one that had satisfying performances for both these features of clustering methods in all the scenarios investigated. SHIPS did not fail to uncover the structure in simulated datasets like Admixture and PCAclust and did not miss the fine complex separation of the populations in real datasets like Structure or AWclust.

In terms of ease of use of the algorithms, the non-parametric ones generally have the advantage of demanding fewer input parameters than parametric approaches. In addition to the data, SHIPS needs the maximal number of clusters investigated and the number of null simulations for the gap statistics. Usually parametric algorithms need a lot of input parameters, often pertaining to the underlying statistical models and therefore more complicated to set. This is the case of Structure, however Admixture needs only the maximal number of clusters and the parameter to conduct the cross-validation.

Considering the computation time of the algorithms, PCAclust is the faster, e.g taking less than an hour when applied to the Pan-Asian data. SHIPS and Admixture take a couple of hours while AWclust is close to a day and Structure several days. Even though PCAclust is the fastest algorithm that we considered in our comparison, one has to note that the program does not come as a package and has to be recoded. The other methods that we considered have the advantage of being freely available in the form of packages.

Several particularities of the SHIPS algorithm can be highlighted. The divisive strategy is based on the rationale that a clustering method has to be applied iteratively to the sub-populations in order to detect the cryptic structures that are hidden behind the main structure of the data. SHIPS finely investigates each estimated cluster to determine if it can be divided into several relevant sub-clusters. This division procedure, that is equivalent to the construction of a binary tree, is conducted by the use of a spectral clustering that takes as input a similarity matrix. This similarity matrix has to be computed only once for all the data and sub-matrices corresponding to the sub-clusters investigated can be extracted at each step. This renders the construction of the tree a fast and efficient part of the algorithm. One has to note that the individual assignment part of the SHIPS algorithm is intimately linked to the choice of a proper similarity matrix. We decided to consider a matrix based on the allele sharing distance as it is computationally fast to compute and led to accurate clustering results. It is however possible to use different matrices that could lead to even better clustering performances [27]. It has been demonstrated that matrices based solely on the allele sharing distance can have low power for the identification of population structure compared to more elaborate distances taking into account other features of the data such as for instance the dependencies between the markers or the relatedness between the samples. Example of such distances can be found in [25], [26] and could easily be used with SHIPS. Indeed, a flexibility of the SHIPS algorithm is that a large variety of similarity matrices can be used to conduct the sample assignment.

The pruning procedure leads to several possible clusterings of the samples. These configurations are all nested within each other. This allows in one run of the algorithm to get for all possible *K* the corresponding clusterings. This information is useful if the user does not desire to use the estimation procedure of *K* and wants to manually look at the clustering possibilities. The hierarchical clustering of AWclust proposes the same option, while software such as Admixture, Structure or PCAclust have to be applied each time for each possible number of clusters. In addition, this allows a fast application of the gap statistic that needs all clustering options for varying numbers of clusters.

SHIPS does not use the same version of the gap statistic than the one used in AWclust. As explained in the Methods section, we decided not to consider the logarithm of the within-cluster sum of squares but directly the sum of squares. This indicator showed better empirical performances to estimate the optimal *K*. Given that AWclust was sometimes able to infer the structure of certain data when manually setting a value for *K* but that the version of the gap statistic used in the program failed to do so, we are confident in our choice of statistic. This gap statistic is rather precise but, like all gap statistics, a time consuming method to estimate the number of clusters. Certain methods, such as AWclust, therefore limit the maximum number of clusters investigated in order to accelerate the whole clustering process. We decided not to make this limitation in the SHIPS package in order to let the user of the program the choice of a reasonable maximum number of clusters.

Also, we determined through several experiments that repetitive applications of the SHIPS algorithm to the same dataset leads to the same clustering results. This robustness of the algorithm confirms that SHIPS is a powerful tool to detect population structure.

The novel clustering approach presented in this paper was applied to SNP data. It produces accurate clustering results and is therefore a promising method to uncover the genetic structure of many populations. Also, one has to note that the methodology of SHIPS, that is the divisive strategy, the following pruning and the gap statistic can easily be extended to cluster other sorts of data such as gene expression for example. Given that a proper distance matrix is used and that an adequate simulation process for null reference datasets of the gap statistic is applied, various usages of the SHIPS algorithm can be expected.

## Supporting Information

Methods S1 **A detailed description of all the clustering algorithms included in the comparison.**
(PDF)

Methods S2 **The complete Genome commands and models used to simulate the data.**
(PDF)

Table S1 **Details of the simulated datasets.**
(PDF)

Table S2 **Details of the admixed dataset.**
(PDF)

Table S3 **Details of the HapMap datasets.**
(PDF)

Table S4 **Details of the Pan-Asian datasets.**
(PDF)

Table S5 **Numbers of Principal components selected by the Tracy-Widom statistic for the PCAclust method.**
(PDF)

Figure S1 **Graphical output of SHIPS representing the estimation of** ***K*** **for the model M1.** The first replicate of the small data was used to produce this plot.
(PDF)

Figure S2 **Graphical output of SHIPS representing the estimation of** ***K*** **for the model M3.** The first replicate of the small data was used to produce this plot.
(PDF)

Figure S3 **Graphical output of SHIPS representing the estimation of** ***K*** **for the model M5.** The first replicate of the small data was used to produce this plot.
(PDF)

Figure S4 **Graphical output of SHIPS representing the estimation of** ***K*** **for the model M10.** The first replicate of the small data was used to produce this plot.
(PDF)

Figure S5 **Graphical output of SHIPS representing the estimation of** ***K*** **for the model M20.** The first replicate of the small data was used to produce this plot.
(PDF)

Figure S6 **Graphical output of SHIPS representing the estimation of** ***K*** **for the admixed data.** The first replicate of the small data was used to produce this plot.
(PDF)

Figure S7 **Graphical output of SHIPS representing the estimation of** ***K*** **for the HapMap data.** The first replicate of the small data was used to produce this plot.
(PDF)

Figure S8 **Graphical output of SHIPS representing the estimation of** ***K*** **for the Pan-Asian data.** The first replicate of the small data was used to produce this plot.
(PDF)

Figure S9 **Graphical output of the SHIPS tree for the model M3 on the small data.** The first replicate of the small data was used to produce this plot.
(PDF)

Figure S10 **Graphical output of the SHIPS tree for the model M5 on the small data.** The first replicate of the small data was used to produce this plot.
(PDF)

Figure S11 **Graphical output of the SHIPS tree for the model M10 on the small data.** The first replicate of the small data was used to produce this plot.
(PDF)

Figure S12 **Graphical output of the SHIPS tree for the model M20 on the small data.** The first replicate of the small data was used to produce this plot.
(PDF)

Figure S13 **Graphical output of the SHIPS tree for the model M3 on the large data.**
(PDF)

Figure S14 **Graphical output of the SHIPS tree for the model M5 on the large data.**
(PDF)

Figure S15 **Graphical output of the SHIPS tree for the model M10 on the large data.**
(PDF)

Figure S16 **Graphical output of the SHIPS tree for the model M20 on the large data.**
(PDF)

Figure S17 **Graphical output of the SHIPS tree for the large HapMap dataset.**
(PDF)

Figure S18 **Graphical output of the SHIPS tree for the large Pan-Asian dataset.**
(PDF)

Figure S19 **Admixture proportions of the different methods for the large admixed dataset (model Madx).** Populations are separated by black lines and assigned with a unique color that is approximatively reported on the barplot of each method. For the discrete methods the admixture proportions are either 0 or 1.
(PDF)

Figure S20 **Admixture proportions of the different method for the large HapMap dataset.** Populations are separated by black lines and assigned with a unique color that is approximatively reported on the barplot of each method. For the discrete methods the admixture proportions are either 0 or 1.
(PDF)

Figure S21 **Admixture proportions of the different method for the large Pan-Asian dataset.** Populations are separated by black lines and assigned with a unique color that is approximatively reported on the barplot of each method. For the discrete methods the admixture proportions are either 0 or 1.
(PDF)
