# Supplementary material for: SHIPS: Spectral Hierarchical Clustering for the Inference of Population Structure in Genetic Studies
Source: PLoS One. 2012 Oct 12;7(10):e45685. doi: 10.1371/journal.pone.0045685 (PMC3470591; doi:10.1371/journal.pone.0045685)
Supplement: Methods S1 — A detailed description of all the clustering algorithms included in the comparison. (PDF) [file pone.0045685.s001.pdf]

Method S1: Methodological details of the clustering algorithms included in the comparison

Contents

1 Structure [1–3] **2**

1.1 The model without admixture 2

1.2 The model with admixture 2

1.3 Estimation of the number of clusters 2

2 Admixture **3**

2.1 Statistical model 3

2.2 Estimation of the number of clusters 3

3 AWclust [4, 5] **3**

4 PCAclust [6] **4**

1 Structure [1–3]

`Structure` is a parametric algorithm that uses Bayesian statistical inference to cluster individuals from genotype data or to determine admixture proportions[1]. Different statistical models are associated with these two endgames. Both models make the Hardy-Weinberg assumption on the SNP data. The other assumptions on the data are the distribution indicated hereafter.

1.1 The model without admixture

Let K be the number of sub-populations from which were sampled the n individuals, $Z = (Z_1, ..., Z_n)$ the unknown vector of population labels and $P = (p_{kjl})_{\substack{1 \leqslant k \leqslant K \\ 1 \leqslant j \leqslant p \\ 1 \leqslant l \leqslant 2}}$ the frequency of allele l at locus j in population k. X represents here the genotype matrix of bi-allelic unlinked markers.
Bayesian inference is used to obtain the distribution of $Pr(Z, P \mid X)$. This is done using the posterior distribution

$$Pr(Z, P \mid X) \propto Pr(Z)Pr(P)Pr(X \mid Z, P),$$

where an uniform prior is chosen for Z and a Dirichlet prior for P

$$\forall\ i = 1 \dots n,\ \forall\ k = 1 \dots K,\ P(Z_i = k) = 1/K,$$

$$\forall\ k = 1 \dots K,\ \forall\ j = 1 \dots p,\ \forall\ l = 0 \dots 2,\ p_{kjl} \sim \mathcal{D}(\lambda_1, \lambda_2),$$

and $Pr(X \mid Z, P)$ is given by

$$\forall\ i = 1 \dots n,\ j = 1 \dots p,\ \forall\ l = 0 \dots 2,\ Pr(x_{ij}^{(a)} = l \mid Z, P) \text{ is defined by } p_{z_i jl},$$

where $x_{ij}^{(a)}$ is the copy of allele a for sample i at locus j.

The posterior distribution $Pr(Z, P \mid X)$ cannot be computed exactly but observations $(Z^{(1)}, P^{(1)})$, ..., $(Z^{(M)}, P^{(M)})$ can be approximated using Markov Chain Monte Carlo (MCMC) estimations.

1.2 The model with admixture

To account for admixture, a new parameter is introduced in the model. The parameter $Q = (q_{ik})_{\substack{1 \leqslant i \leqslant n \\ 1 \leqslant k \leqslant K}}$ represents the proportion of individual i's genome that originated from population k. For each individual, this parameter follows a Dirichlet prior distribution as well. The quantity that is estimated with MCMC simulations are then $(Z^{(1)}, P^{(1)}, Q^{(1)})$, ..., $(Z^{(M)}, P^{(M)}, Q^{(M)})$.

1.3 Estimation of the number of clusters

The two models previously introduced allow one to estimate the populations of origin of the individuals in the case of a known number of populations K. In practice, this number is unknown and has to be estimated. Prithcard et al. propose a way of estimating K using a Bayesian approach, therefore the posterior distribution

$$Pr(K \mid X) \propto Pr(X \mid K)Pr(K).$$

Problems arise to compute $Pr(X \mid K)$ reason why they also propose an *had oc* solution that is selecting the number of clusters K maximizing an estimation of $log(Pr(X|K))$. This information is given in the output of the program along with the admixture proportions or the population labels.

---

[1] i.e the proportion of each individual's genome that comes from each of the estimated sub-populations.

Recent algorithms such as Structurama [7] allow a better estimation of K and can be combined with the clusterings estimated by Structure to provide more accurate estimations of the populations ancestries. Due to computational limitations we were not able to run Structurama and therefore decided to opt for a way to estimate the number of clusters with Structure that advantages the method. In our comparison strategy a criterion is used to compare the different programs and we considered an estimated K for Structure that optimizes this criterion.

2 Admixture

2.1 Statistical model

`Admixture` is another very popular parametric algorithm that estimates admixture proportions [8, 9]. `Admixture` uses the same statistical model as `Structure` however instead of sampling priors using MCMC estimations, the program directly maximizes the likelihood. `Admixture` assumes the Hardy-Weinberg equilibrium between the unlinked markers.

To estimate the parameters Q and P, the algorithm maximizes the log-likelihood

$$L(Q,P) = \sum_{i,j} \{x_{ij} ln(f_{ij}) + (2 - x_{ij} ln(1 - f_{ij}))\},$$

where $f_{ij} = \sum_{k=1}^{K} q_{ik} p_{kj1}$.

In order to accelerate the estimation several statistical techniques are employed. A block relaxation algorithm is used to conduct the optimization. The convergence of this algorithm is accelerated using a quasi-Newton method and the standard-errors of the parameters are calculated using a moving block bootstrap method.

2.2 Estimation of the number of clusters

The estimation of the number of clusters K is conducted by a cross-validation technique. This method partitions the initial data into several subsets and uses these subsets to validate the estimated K.

3 AWclust [4, 5]

The `AWclust` (Allele sharing distance and Ward's minimum of variance hierarchical clustering) algorithm is composed of three steps.

1. A distance matrix between all pairs of individuals is computed. This is a dissimilarity matrix based on the allele sharing distance. The dissimilarity at SNP k between samples i et j is

$$s_{i,j}(k) = \begin{cases} 0 & \text{if same genotype} \\ 1 & \text{if one common allele} \\ 2 & \text{if no common allele} \end{cases}$$

2. A hierarchical clustering is applied to the distance matrix. Initially each individual forms a single cluster. The clusters are progressively merged following Ward's minimum of variance criterion until all samples are in the same cluster.

3. The estimation of the number of clusters K is made using the gap statistic. Contrary to the version of the gap statistic used in SHIPS the quality criterion of each possible clustering $C_k = (D_1, \ldots, D_k)$ with k clusters is
$$log(W_k) = log(\sum_{r=1}^{k} \frac{1}{2}.\Sigma(D_r)),$$
where $\Sigma(D_r)$ is the sum of the squared dissimilarities within the $r - th$ class of C_k.

   Note that the AWclust algorithm limits the maximum number of clusters to 16 in order to reduce the computational cost of the method.

4 PCAclust [6]

The PCAclust algorithm we considered in the comparison uses the software EIGENSOFT 3.0 developed by Patterson et al. [10,11] and R. This method is composed of the 3 following steps.

1. A principal component analysis is applied to the genotype data to define new variables that are the principal components (PCs). This implies a singular vector decomposition of the genotype covariance matrix XX'. This step is conducted using Eigensoft 3.0 using the LD option that replaces each SNP by the residual of its regression on the two preceding SNPs.
2. A set of significant PCs, $(PC_1,..,PC_m)$, is selected using the Tracy-Widom statistics at a 5% level.
3. A Gaussian Mixture Model (GMM) clustering algorithm is applied to the selected PCs to cluster the samples. The estimated number of clusters is computed so that the likelihood of the model is maximized. the R package Mclust is used for this clustering.

References

1. Pritchard JK, Stephens M, Donnelly P (2000) Inference of population structure using multilocus genotype data. Genetics 155: 945–959.

2. Falush D, Stephens M, Pritchard JK (2003) Inference of population structure using multilocus genotype data: linked loci and correlated allele frequencies. Genetics 164: 1567–1587.

3. Falush D, Stephens M, Pritchard JK (2007) Inference of population structure using multilocus genotype data: dominant markers and null alleles. Mol Ecol Notes 7: 574–578.

4. Gao X, Starmer J (2007) Human population structure detection via multilocus genotype clustering. BMC Genet 8: 34.

5. Gao X, Starmer JD (2008) Awclust: point-and-click software for non-parametric population structure analysis. BMC Bioinformatics 9: 77.

6. Lee C, Abdool A, Huang CH (2009) Pca-based population structure inference with generic clustering algorithms. BMC Bioinformatics 10 Suppl 1: S73.

7. Huelsenbeck JP, Andolfatto P (2007) Inference of population structure under a dirichlet process model. Genetics 175: 1787–1802.

8. Alexander DH, Novembre J, Lange K (2009) Fast model-based estimation of ancestry in unrelated individuals. Genome Res 19: 1655–1664.

9. Alexander DH, Lange K (2011) Enhancements to the admixture algorithm for individual ancestry estimation. BMC Bioinformatics 12: 246.

10. Patterson N, Price AL, Reich D (2006) Population structure and eigenanalysis. PLoS Genet 2: e190.

11. Price AL, Patterson NJ, Plenge RM, Weinblatt ME, Shadick NA, et al. (2006) Principal components analysis corrects for stratification in genome-wide association studies. Nat Genet 38: 904–909.
