# Supplementary material for: SHIPS: Spectral Hierarchical Clustering for the Inference of Population Structure in Genetic Studies
Source: PLoS One. 2012 Oct 12;7(10):e45685. doi: 10.1371/journal.pone.0045685 (PMC3470591; doi:10.1371/journal.pone.0045685)
Supplement: Methods S2 — The complete Genome commands and models used to simulate the data. (PDF) [file pone.0045685.s002.pdf]

Population histories and GENOME commands for the simulated populations

Model M1

```
genome -pop 1 100 -c 20 -s 250
```

Model M3

```
genome -pop 3 100 100 100 -c 20 -s 250 -N model3.txt
```

with the population history model3.txt

0 100 100 100
1-1 2-2 3-2
10 1000 1000
1-1 2-2
20 1000 1000

Model M5

```
genome -pop 5 50 50 50 50 50 -c 20 -s 250 -N model5.txt
```

with the population history model5.txt

0 50 50 50 50 50
1-1 2-1 3-1 4-2 5-2
10 1000 1000
1-1 2-2
20 1000 1000

Model M10

```
genome -pop 10 50 50 50 50 50 50 50 50 50 50 -c 20 -s 250 -N model10.txt
```

with the population history model10.txt

0 50 50 50 50 50 50 50 50 50 50
1-1 2-2 3-2 4-3 5-4 6-5 7-5 8-6 9-7 10-7
30 1000 1000 1000 1000 1000 1000 1000
1-1 2-2 3-2 4-3 5-3 6-4 7-4
70 1000 1000 1000 1000
1-1 2-1 3-2 4-3

100 1000 1000 1000

Model M20

```
genome -pop 20 30 30 30 30 30 30 30 30 30 30 30 30 30 30 30 30 30 30 30 30 -c 20
 -s 250 -N model20.txt
```

with the population history model20.txt

0 30 30 30 30 30 30 30 30 30 30 30 30 30 30 30 30 30 30 30 30
1-1 2-1 3-1 4-2 5-2 6-3 7-3 8-3 9-4 10-4 11-5 12-5 13-5 14-6 15-6 16-7 17-7 18-8 19-9 20-10
30 1000 1000 1000 1000 1000 1000 1000 1000 1000 1000
1-1 2-1 3-2 4-2 5-3 6-3 7-4 8-4 9-5 10-6
70 1000 1000 1000 1000 1000 1000
1-1 2-1 3-2 4-2 5-3 6-3
100 1000 1000 1000
