# Supplementary material for: SHIPS: Spectral Hierarchical Clustering for the Inference of Population Structure in Genetic Studies
Source: PLoS One. 2012 Oct 12;7(10):e45685. doi: 10.1371/journal.pone.0045685 (PMC3470591; doi:10.1371/journal.pone.0045685)
Supplement: Table S1 — Details of the simulated datasets. (PDF) [file pone.0045685.s003.pdf]

Model	Samples per sub-population
Model M1 (1 sub-population)	100
Model M3 (3 sub-populations)	100
Model M5 (5 sub-populations)	50
Model M10 (10 sub-populations)	50
Model M20 (20 sub-populations)	30

Details of the simulated models
