# Supplementary material for: SHIPS: Spectral Hierarchical Clustering for the Inference of Population Structure in Genetic Studies
Source: PLoS One. 2012 Oct 12;7(10):e45685. doi: 10.1371/journal.pone.0045685 (PMC3470591; doi:10.1371/journal.pone.0045685)
Supplement: Table S2 — Details of the admixed dataset. (PDF) [file pone.0045685.s004.pdf]

Population	# Samples
CEU	112
CHB	137
Admixed (names XY)	100

Details of the admixed dataset
