# Supplementary material for: SHIPS: Spectral Hierarchical Clustering for the Inference of Population Structure in Genetic Studies
Source: PLoS One. 2012 Oct 12;7(10):e45685. doi: 10.1371/journal.pone.0045685 (PMC3470591; doi:10.1371/journal.pone.0045685)
Supplement: Table S3 — Details of the HapMap datasets. (PDF) [file pone.0045685.s005.pdf]

Population	Ethnicity	# Samples
CEU	Utah residents with Northern and Western European ancestry	112
CHB	Han Chinese in Beijing, China	137
CHD	Chinese in Metropolitan Denver, Colorado	109
GIH	Gujarati Indians in Houston, Texas	101
JPT	Japanese in Tokyo, Japan	113
LWK	Luhya in Webuye, Kenya	110
MKK	Maasai in Kinyawa, Kenya	156
TSI	Toscani in Italia	102
YRI	Yoruba in Ibadan, Nigeria	147

Details of the HapMap dataset
