# Supplementary material for: SHIPS: Spectral Hierarchical Clustering for the Inference of Population Structure in Genetic Studies
Source: PLoS One. 2012 Oct 12;7(10):e45685. doi: 10.1371/journal.pone.0045685 (PMC3470591; doi:10.1371/journal.pone.0045685)
Supplement: Table S4 — Details of the Pan-Asian datasets. (PDF) [file pone.0045685.s006.pdf]

Population	Ethnicity	# Samples
CN.WA	Wa, China	56
ID.JA	Javanese, Indonesia	34
IN.TB	Mongoloid features, India	23
JP.ML	Japanese ,Japan	71
KR.KR	Koreans, Korea	90
MY.JH	Negrito, Malaysia	50
PI.AT	Ati, Philippines	23
SG.ID	Indian, Singapore	30
TH.MA	Mlabri, Thailand	18
TW.HA	Chinese, Taiwan	48

Details of the Pan-Asian dataset
