# Supplementary material for: SHIPS: Spectral Hierarchical Clustering for the Inference of Population Structure in Genetic Studies
Source: PLoS One. 2012 Oct 12;7(10):e45685. doi: 10.1371/journal.pone.0045685 (PMC3470591; doi:10.1371/journal.pone.0045685)
Supplement: Table S5 — Numbers of Principal components selected by the Tracy-Widom statistic for the PCAclust method. (PDF) [file pone.0045685.s007.pdf]

Data / Model	M1	M3	M5	M10	M20	Madx	HapMap	Pan-Asian
Small data	0	17	12	51	72	9	49	64
Large data	0	6	5	28	49	25	70	99

Number of PCs selected with PCAclust
