# Supplementary material for: SHIPS: Spectral Hierarchical Clustering for the Inference of Population Structure in Genetic Studies
Source: PLoS One. 2012 Oct 12;7(10):e45685. doi: 10.1371/journal.pone.0045685 (PMC3470591; doi:10.1371/journal.pone.0045685)
Supplement: Figure S8 — Graphical output of SHIPS representing the estimation of K for the Pan-Asian data. The first replicate of the small data was used to produce this plot. (PDF) [file pone.0045685.s015.pdf]

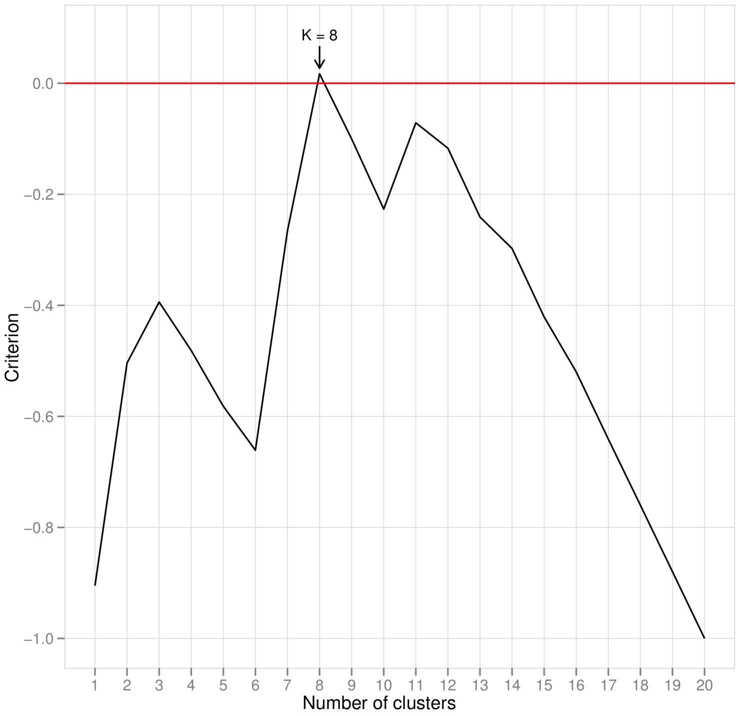

Estimation of the number of clusters with SHIPS (Pan-Asian). Representation of the criterion used to determine the estimated number of clusters $crit(k) = Gap(k) - Gap(\tilde{k}) + s_{\tilde{k}}$. The estimated K is the smallest k such as $crit(k) \geq 0$.
The black line represents the criterion and the red line the 0 threshold.
The first replicate was used to produce this plot.
