# Supplementary material for: SHIPS: Spectral Hierarchical Clustering for the Inference of Population Structure in Genetic Studies
Source: PLoS One. 2012 Oct 12;7(10):e45685. doi: 10.1371/journal.pone.0045685 (PMC3470591; doi:10.1371/journal.pone.0045685)
Supplement: Figure S9 — Graphical output of the SHIPS tree for the model M3 on the small data. The first replicate of the small data was used to produce this plot. (PDF) [file pone.0045685.s016.pdf]

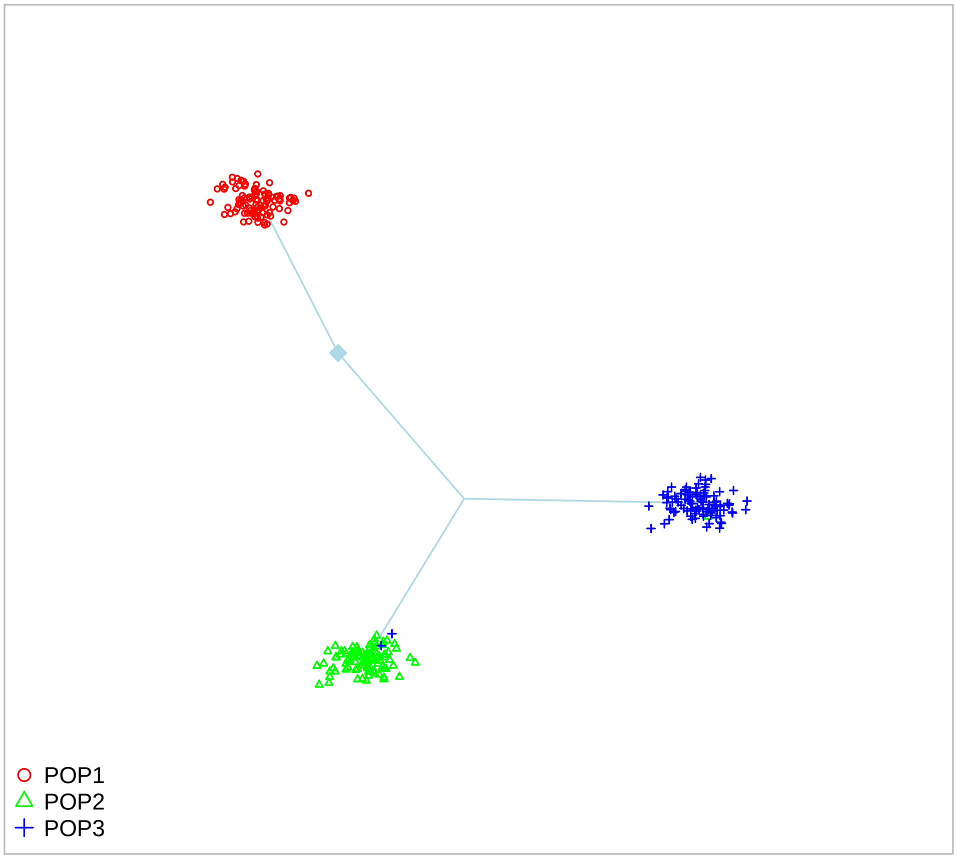

SHIPS tree of the model M3 (small dataset) This plot is obtained from the first small dataset. The colored populations correspond to the population labels and not the estimated clusters.
