# Supplementary material for: SHIPS: Spectral Hierarchical Clustering for the Inference of Population Structure in Genetic Studies
Source: PLoS One. 2012 Oct 12;7(10):e45685. doi: 10.1371/journal.pone.0045685 (PMC3470591; doi:10.1371/journal.pone.0045685)
Supplement: Figure S18 — Graphical output of the SHIPS tree for the large Pan-Asian dataset. (PDF) [file pone.0045685.s025.pdf]

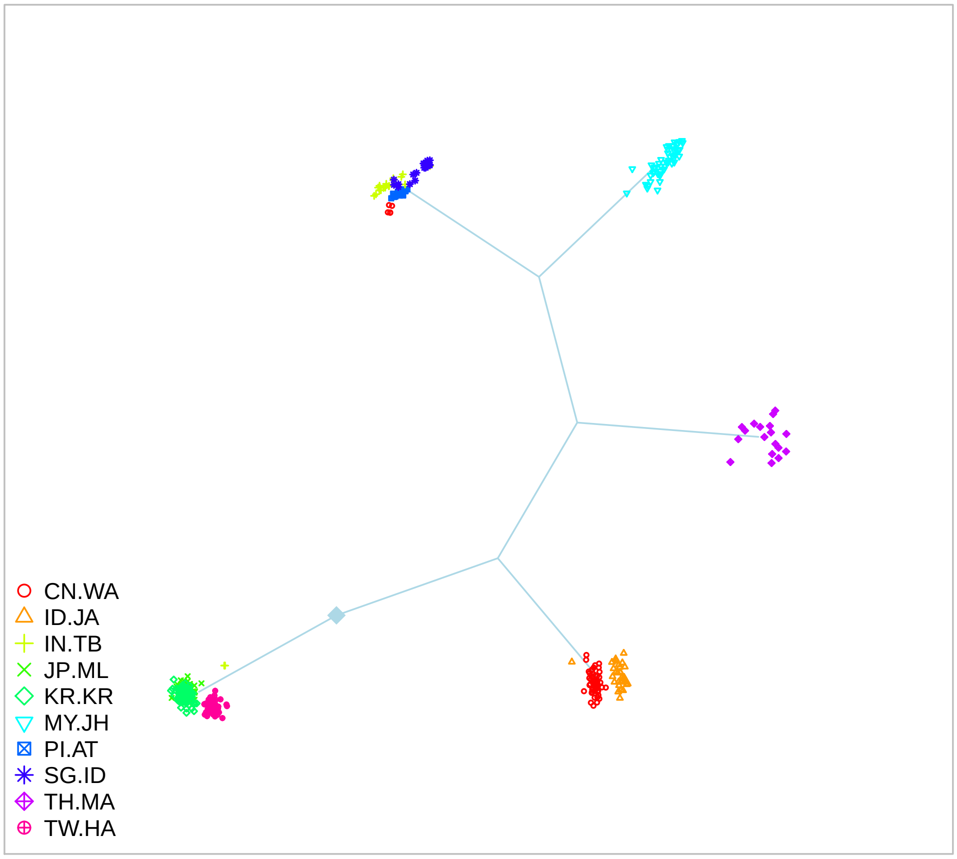

SHIPS tree of the large Pan-Asian dataset (K=10) The colored populations correspond to the population labels and not the estimated clusters.
