# Supplementary material for: SHIPS: Spectral Hierarchical Clustering for the Inference of Population Structure in Genetic Studies
Source: PLoS One. 2012 Oct 12;7(10):e45685. doi: 10.1371/journal.pone.0045685 (PMC3470591; doi:10.1371/journal.pone.0045685)
Supplement: Figure S20 — Admixture proportions of the different method for the large HapMap dataset. Populations are separated by black lines and assigned with a unique color that is approximatively reported on the barplot of each method. For the discrete methods the admixture proportions are either 0 or 1. (PDF) [file pone.0045685.s027.pdf]

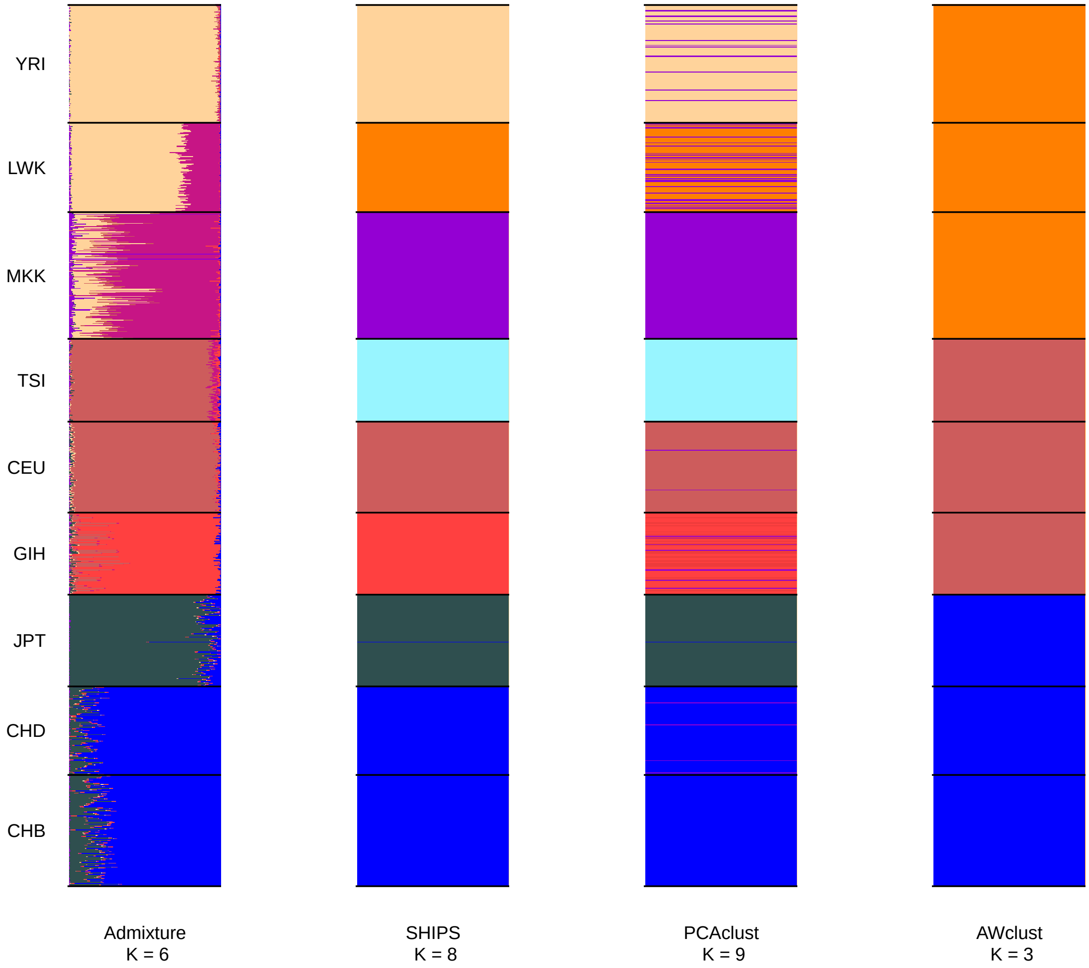


**Barplots of the admixture proportions for the large HapMap data.** Populations are separated by black lines and assigned with a unique color that is approximatively reported on the barplot of each method. For the discrete methods the admixture proportions are either 0 or 1.
